# Nicotinamide mononucleotide ameliorates adriamycin-induced renal damage by epigenetically suppressing the NMN/NAD consumers mediated by Twist2

**DOI:** 10.1038/s41598-022-18147-2

**Published:** 2022-08-12

**Authors:** Kazuhiro Hasegawa, Yusuke Sakamaki, Masanori Tamaki, Shu Wakino

**Affiliations:** 1 Department of Nephrology, Tokushima University Graduate School of Biomedical Sciences, 3-18-15 Kuramoto-cho, Tokushima, 770-8503 Japan; 2 Department of Internal Medicine, Tokyo Dental College Ichikawa General Hospital, Chiba, 272-8583 Japan

**Keywords:** Chronic kidney disease, Glomerular diseases

## Abstract

The activation of nicotinamide adenine dinucleotide (NAD^+^)-dependent deacetylase, Sirt1, after the administration of nicotinamide mononucleotide (NMN) suppresses many diseases. However, the role of NMN and Sirt1 in focal glomerulosclerosis (FSGS) has not yet been elucidated. This study aimed to assess the protective effect of NMN treatment in mice with adriamycin (ADR)-induced FSGS.　Transient short-term NMN treatment was administered to 8-week-old ADR- or saline-treated BALB/c mice (Cont group) for 14 consecutive days. NMN alleviated the increase in urinary albumin excretion in the ADR-treated mice. NMN treatment mitigated glomerulosclerosis and ameliorated the reduced Sirt1 expression and elevated Claudin-1 expression in the kidneys of the mice. Moreover, this treatment improved the decrease in histone methylation and the expression level of Dnmt1 and increased the concentration of NAD^+^ in the kidney. Dnmt1 epigenetically suppressed the expression of the NMN-consuming enzyme nicotinamide mononucleotide adenyltransferase1 (Nmnat1) by methylating the E-box in the promoter region and repressing the NAD-consuming enzyme PARP1. Additionally, NMN downregulated the expression of Nmnat1 in the ADR-treated mice. Short-term NMN treatment in FSGS has epigenetic renal protective effects through the upregulation of Sirt1 and suppression of the NAD and NMN consumers. The present study presents a novel treatment paradigm for FSGS.

## Introduction

Adriamycin (ADR)-induced nephropathy is a murine model of human focal glomerulosclerosis (FSGS), which is characterized by podocyte damage, glomerular sclerosis, and tubulointerstitial fibrosis^1^. In another study, podocyte conditional Sirt1 knockout resulted in aggravated podocyte injury after aldosterone infusion in mice. Lu reported that ADR-induced FSGS models presented with podocyte injury due to the downregulation of Sirt1^2^. Nonetheless, whether Sirt1 can rescue FSGS-induced podocyte injury is unknown. We previously demonstrated that Sirt1 knockout in proximal tubular cells decreased its expression in glomerular podocytes and increased the expression of a tight junction protein, Claudin-1, which resulted in albuminuria^3^.

FSGS is the leading cause of end-stage renal disease^1^. In a previous study, we demonstrated that Sirt1 inactivation in podocytes upregulated the ectopic expression of Claudin-1, leading to the abrogation of glomerular barrier function via epigenetic mechanisms (reduced methylation of the Claudin-1 gene)^3^. In an RNA sequence analysis using human FSGS samples^4^, Claudin-1 ectopic overexpression in the podocyte was reportedly correlated with podocyte damage. Transgenic mice with Claudin-1 overexpression in the podocytes demonstrated both podocyte injury and proteinuria^5^. Sirt1 exerts its effects via the protein deacetylase activity and the histone deacetylase activity. It regulates the expression levels of Claudin-1^3^ and various other genes, epigenetically, via the histone deacetylation activity, along with DNA methylation. The upregulation of Claudin-1 might lead to glomerular damage, considering that the epigenetic effects could last for a prolonged period. This gene regulation effect is thought to be involved in the memory or legacy effects observed in diabetic complications, which have been observed in a previous large clinical trial (UKPDS80)^6^.

**Figure 1:**
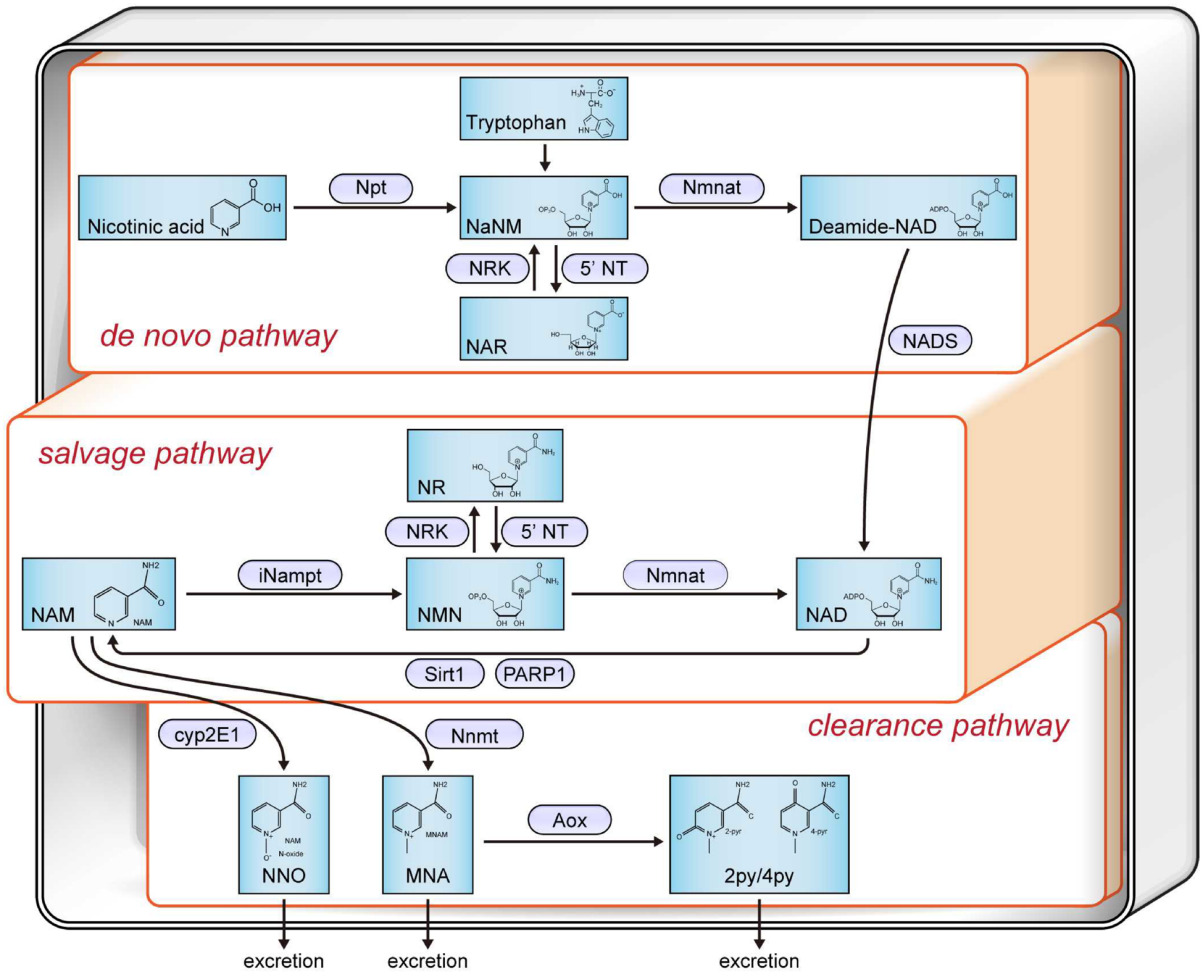
Schematic diagram showing the NAD^+^ metabolic pathway. *Npt* nicotinic acid phosphoribosyltransferase, *Nmnat* nicotinamide mononucleotide adenylyl transferase, *NRK* nicotinamide riboside kinase, *5′-NT* 5′-nucleotidase, *iNampt* intracellular NAM phosphoribosyl transferase, *Sirt1* Sirtuin1, *Cyp2E1* Cytochrome P450 2E1, *Nnmt* nicotinamide N-Methyltransferase, *Aox* aldehyde oxidase, *NaNM* nicotinic acid mononucleotide, *NAR* nicotinic acid riboside, *NAM* nicotinamide, *NMN* nicotinamide mononucleotide, *NR* nicotinamide riboside, *NAD* nicotinamide adenine dinucleotide, *NNO* NAM N-oxide, *MNA* N1-methylniacinamide, *2py* N1-methyl-2-pyridone-5-carboxamide, *4py* N1-methyl-4-pyridone-3-carboxamide, *PARP1* poly ADP-ribose polymerase 1.

Sirt1 activity depends on the cellular levels of NAD^+^. NAD^+^ concentrations in each organ have been reported to decrease with age and chronic organ damage, which includes CKD or nephrosis in murine models^7,8^. Therefore, increases in NAD levels could be used as potential therapeutic targets in these diseases^9^. Several strategies to increase Sirt1 activity^10^, including caloric restriction^11^, administration of resveratrol^12^, and approaches to restore NAD^+^ levels (such as the administration of NAD^+^ metabolites^13^, or the inhibition of NAD consumers), have been reported^14^. In terms of supplementation with NAD^+^ metabolites to increase the NAD^+^ concentration and Sirt1 activity or expression, several substances such as nicotinamide (NAM) riboside (NR), NAM mononucleotide (NMN), and NAM have been reported to have fewer adverse effects and efficiently enhance NAD^+^ biosynthesis^15–18^. However, although the effects of NMN in cisplatin-induced AKI murine and diabetic nephropathy murine models have been published^19–22^, they have not been tested in the ADR-induced FSGS model thus far. NMN is an intermediate of the NAD^+^ salvage pathway produced by nicotinamide phosphoribosyltransferase (Nampt) from NAM. In this pathway, NMN is further converted to NAD^+^ by nicotinamide mononucleotide adenylyltransferase (Nmnat), which is then recycled in various metabolic and cellular reactions (Fig. 1).

This study aimed to assess the renoprotective effect of preemptive short-term NMN treatment in mice with ADR-induced FSGS. The results suggested that NAD^+^ and Sirt1 deficit contribute to kidney damage susceptibility. Short-term NMN treatment rescued the FSGS from podocyte damage through the restoration of renal NAD^+^ concentrations, even after the termination of the treatment. Additionally, we observed long-term effects of the dynamics of NAD^+^ metabolites after the treatment, suggesting legacy effects by the reactivation of Sirt1. The present study presents a novel treatment paradigm for FSGS, which could increase the possibility of achieving remission in this model.

## Results

### Effect of short-term NMN treatment on kidney function

**Figure2:**
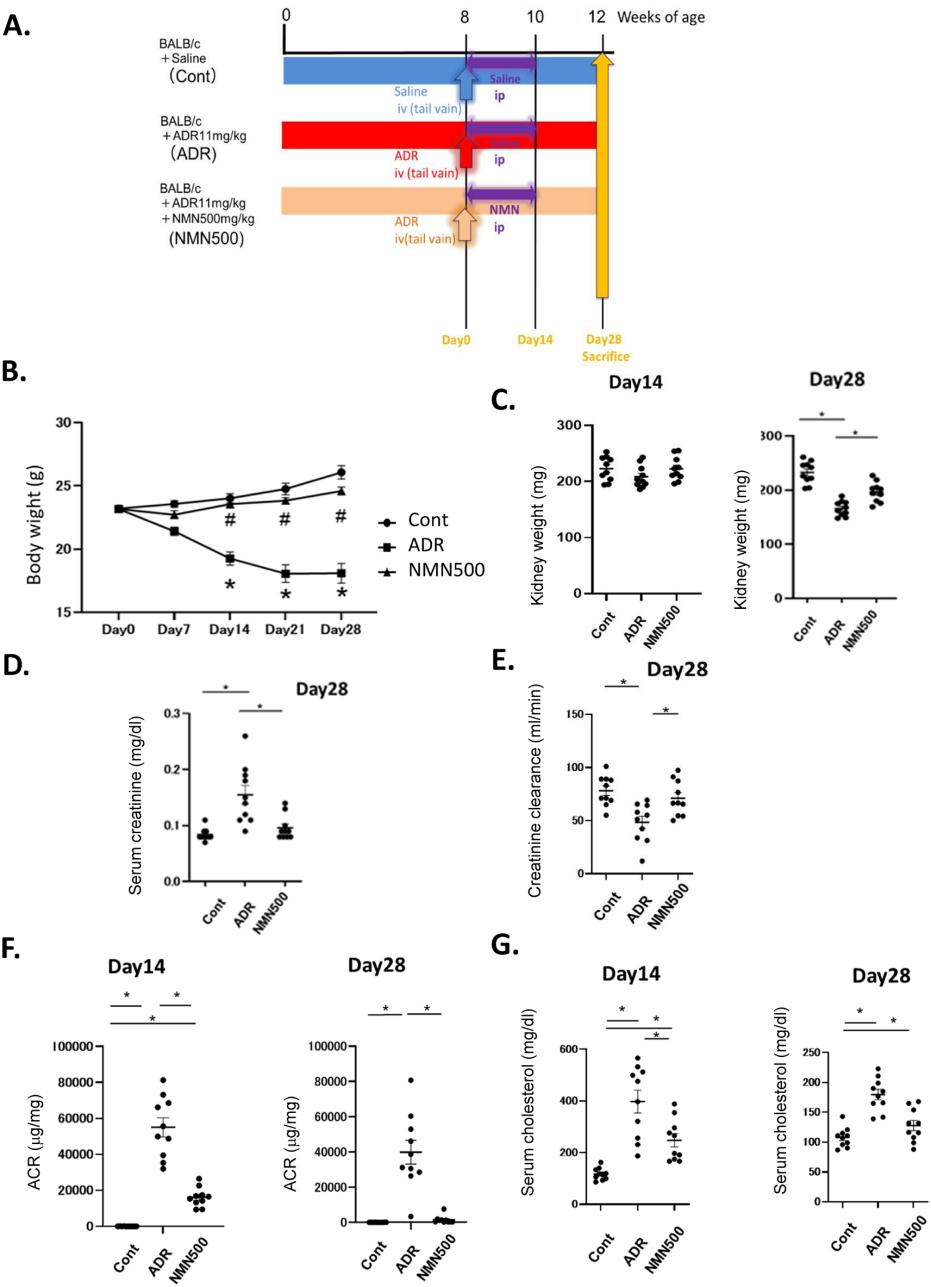
Effect of short-term NMN treatment on kidney functions and survival. (**A**) Schematic diagram of the NMN treatment protocol. (**B**) Temporal changes in the body weights of the mice in the three groups (Cont, *ADR*, and NMN500; n = 10). *P < 0.05 Cont vs. ADR. ^†^*P* < 0.05 Cont vs. NMN500. (**C**) The weights of the kidneys on days 14 and 28 in the three groups (Cont, *ADR*, and NMN500; *n* = 10). (**D**) Serum creatinine levels were measured on day 28 in the three groups (Cont, *ADR*, and NMN500; *n* = 10). (**E**) Creatinine clearance on day 28 in the three groups (Cont, *ADR*, and NMN500; *n* = 10). (**F**) Urine ACR on day 14 (n = 10) and 28 (n = 10) in the three groups (Cont, *ADR*, and NMN500). (**G**) Serum cholesterol levels on day 14 (n = 10), and 28 (n = 10) in the three groups (Cont, *ADR*, and NMN500).

NMN is an NAD^+^ precursor in the salvage pathway (Fig. 1). Transient short-term NMN treatment was administered to 8-week-old ADR-treated BALB/c mice or saline-treated BALB/c mice (Cont group). The ADR-treated mice were administered intraperitoneally with NMN at a dose of 500 mg/kg/day or with normal saline alone for 14 consecutive days (NMN 500 group or ADR group, respectively, Fig. 2A). The body weights of the mice in the NMN500 and Cont groups were greater than those in the ADR group; the weights of the mice in the Cont and NMN500 groups did not differ significantly (Fig. 2B). On day 14, no significant differences in kidney weights were observed among the three groups, whereas on day 28, the weights in the Cont group and NMN500 group were higher than those in the ADR group (Fig. 2C). On day 28, serum creatinine levels in the ADR group were higher than those in the Cont and NMN500 groups (Fig. 2D). Although a decline in glomerular filtration was observed on day 28 in the ADR group, it was reversed in the NMN500 group (Fig. 2E). Moreover, the ADR group exhibited a significantly higher urinary ACR compared to the Cont group, on days 14 and 28 (Fig. 2F). The NMN500 group exhibited lower albuminuria levels than the ADR group on days 14 and 28, which suggested an inhibitory effect of NMN on albuminuria, and this effect was sustained for 28 days even after the termination of the short-term NMN intervention. Serum cholesterol levels were significantly higher in the ADR group than in the Cont and NMN500 groups on days 14 and 28 (Fig. 2G).

**Figure 3:**
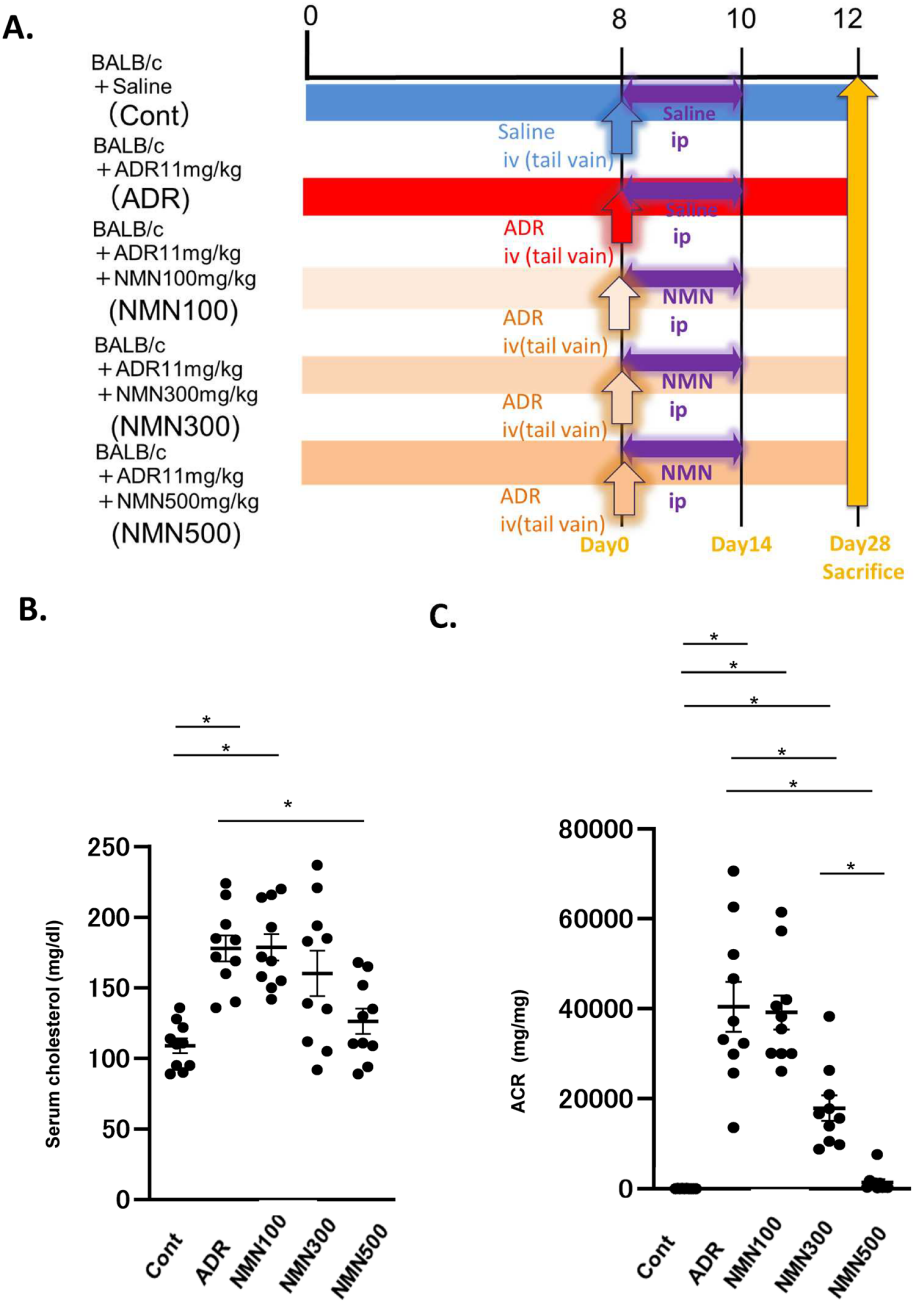
Dose–response study of NMN treatment. (**A**) Schematic diagram illustrating the dose–response study. (**B**) Cholesterol levels on day 28 in the five groups (Cont, *ADR*, NMN100, NMN300, and NMN500; *n* = 15). Statistical significance is represented by an asterisk. *P < 0.05 vs *Cont*. (**C**) Urine ACR on day 28 in the five groups (*Cont*, *ADR*, NMN100, NMN300, and NMN500; *n* = 15). All data are shown as mean ± standard error of the mean. Statistical significance between each group is represented by a horizontal bar. *P < 0.05.

We further examined the dose-dependent effects of short-term NMN treatment (Fig. 3A). The effects of short-term treatment with two additional doses of NMN, 100 and 300 mg/kg, on serum cholesterol levels and urine ACR levels, were evaluated on day 28. The cholesterol levels in ADR mice treated with 100 mg/kg NMN (NMN100) and 300 mg/kg NMN (NMN300) were not different from those in the ADR group (Fig. 3B). Moreover, the NMN300 and NMN500 groups exhibited reduced ACR, whereas the NMN100 group did not show a significant reduction in ACR when compared to the ADR group (Fig. 3C).

### Short-term NMN treatment ameliorated the histological changes in ADR-treated mice

**Figure 4:**
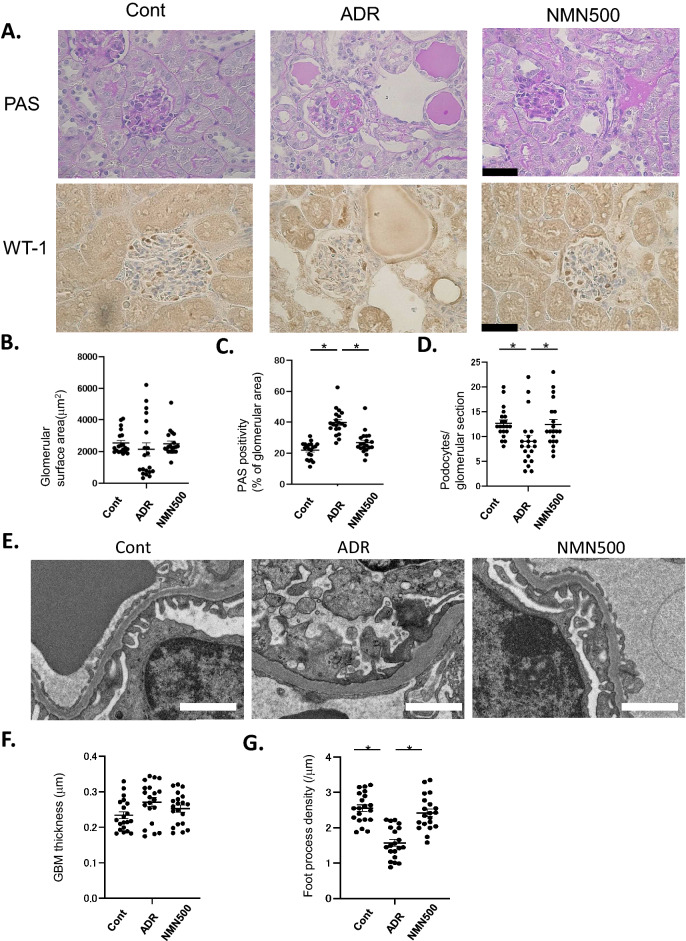
Short-term NMN treatment ameliorated the histological changes in FSGS. (**A**) Representative images of PAS and WT1 immunostaining in the glomeruli of the mice from the Cont, ADR, and NMN500 groups (scale bar 500 µm). (**B**) Graph showing the glomerular surface area. (**C**) Graph showing the PAS positivity in the glomerular area within the PAS-stained kidney sections; *n* = 20 sections per group. (**D**) Graph showing the number of podocytes per glomerulus detected using the antibodies to WT1. (**E**) Representative EM images of GBM in the *Cont*, *ADR*, and NMN500 groups. The GBM and foot processes are indicated (scale bar 1 µm). (**F**) Graph showing the GBM thickness obtained from 20 measurements per group. (**G**) Graph showing the density of the foot process per micron of GBM obtained from approximately 20 measurements per group. All data are shown as mean ± standard error of the mean. Statistical significance between each group is represented by a horizontal bar. *P < 0.05.

To histologically assess the effect of NMN on ADR-induced renal damage, the glomerular volume and mesangial expansion were evaluated via PAS staining and the podocyte number was determined using the podocyte marker WT-1 (Fig. 4A). No significant difference in glomerular surface area was observed among the three groups on day 28 (Fig. 4B). The ADR group exhibited more PAS-positive areas than the Cont group on day 28 (Fig. 4A,C). The PAS-positive areas in the NMN500 group were lower than those in the ADR group on day 28 (Fig. 4C). Furthermore, on day 28, the number of podocytes per glomerular section was lower in the ADR group than that in the Cont group, and this reduction was rescued in the NMN500 group (Fig. 4D). In terms of the EM findings, we investigated the thickness of the GBM and the density of the foot process of the podocytes (Fig. 4E). The GBM thickness did not differ among the Cont, ADR, and NMN500 groups (Fig. 4F), whereas the foot process density was lower in the ADR group than in the Cont group; this reduction was ameliorated in the NMN500 group (Fig. 4G).

### Molecular changes in the glomerulus after NMN treatment

**Figure 5:**
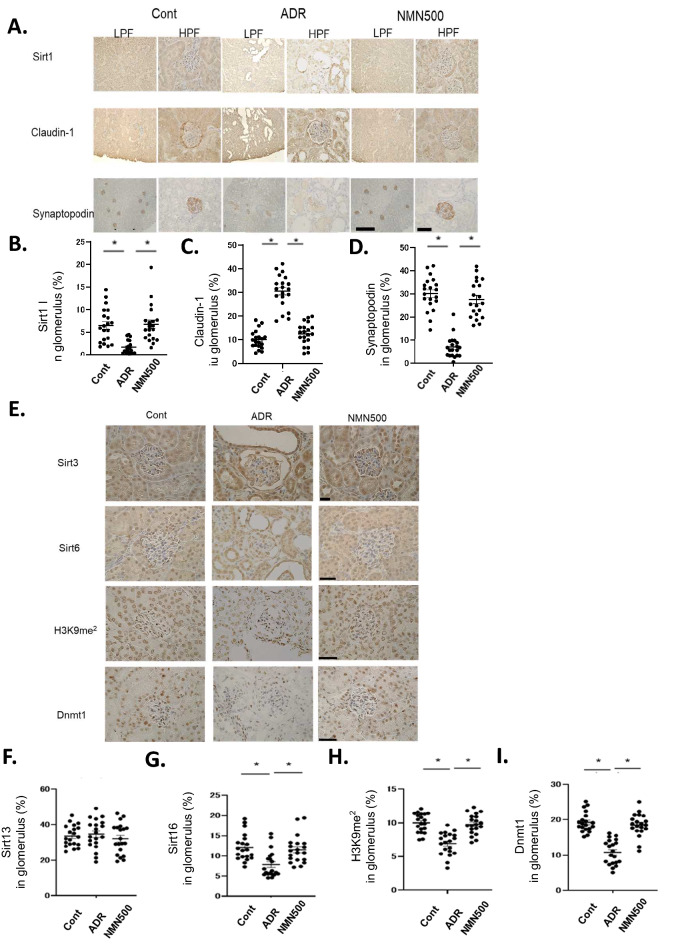
Molecular changes in the glomerulus after NMN treatment. (**A**) Representative images showing immunostaining for Sirt1, Claudin-1, and Synaptopodin in the glomeruli of the animals in the Cont, ADR, and NMN500 groups. Left, low-magnification images (scale bar 50 µm); right, high-magnification images (scale bar 500 µm). (**B–D**) The proportional areas of Sirt1 (**B**), Claudin-1 (**C**), and Synaptopodin (**D)** staining (*n* = 20 sections per group). (**E**) Representative images showing immunostaining for Sirt3, Sirt6, H3K9me2, and Dnmt1 in the glomeruli (scale bar 50 µm). (**F–I**) The proportional areas of Sirt3 (**F**), Sirt6 (**G**), H3K9me2 (**H**), and Dnmt1 (**I**) staining determined by the Image-Pro Plus 7.0J software (*n* = 20 sections per group). All data are shown as mean ± standard error of the mean. Statistical significance between each group is represented by a horizontal bar. *P < 0.05 by ANOVA with Tukey’s post hoc test.

We have previously shown that decreased Sirt1 in podocytes subsequently increases ectopic Claudin-1 expression and causes foot process effacement in the podocyte, leading to diabetic albuminuria^3,22^. In the present study, we assessed the levels of expression of several proteins involved in this mechanism via immunohistochemistry in each mice group on day 28 when NMN treatment was terminated 14 days before (Fig. 5A). Sirt1 expression was decreased in the ADR group compared with the Cont group (Fig. 5B). Claudin-1 expression was increased (Fig. 5C), and Synaptopodin expression was decreased in the ADR group (Fig. 5D) when compared with the Cont group. These changes were ameliorated in the NMN group. We have previously reported that decreased Sirt1 expression causes Claudin-1 expression and podocyte damage through decreased histone H3K9 methylation and decreased Dnmt1 expression in a diabetic glomerular sclerosis background^3,22^. This NMN effect was assessed in the ADR-induced nephropathy model in the present study^23^. To assess other Sirtuin isoforms that are abundantly expressed in the kidney^24^, the expression levels of both Sirt3 and Sirt6 in the glomeruli were determined. No changes in the expressions of Sirt3 were observed between the ADR and NMN500 groups (Fig. 5E,F). Sirt6 expression was decreased in the ADR group and restored in the NMN500 group (Fig. 5E,G). The expression of H3K9me2 was decreased in the ADR group as compared to that in the Cont group, but it was maintained in the NMN500 group (Fig. 5E,H). The expression of Dnmt1 was decreased in the ADR group than in the Cont group; this change was ameliorated in the NMN500 group (Fig. 5E,I).

### Effects of NMN treatment on NAD^+^ metabolites and the salvage pathway

**Figure 6:**
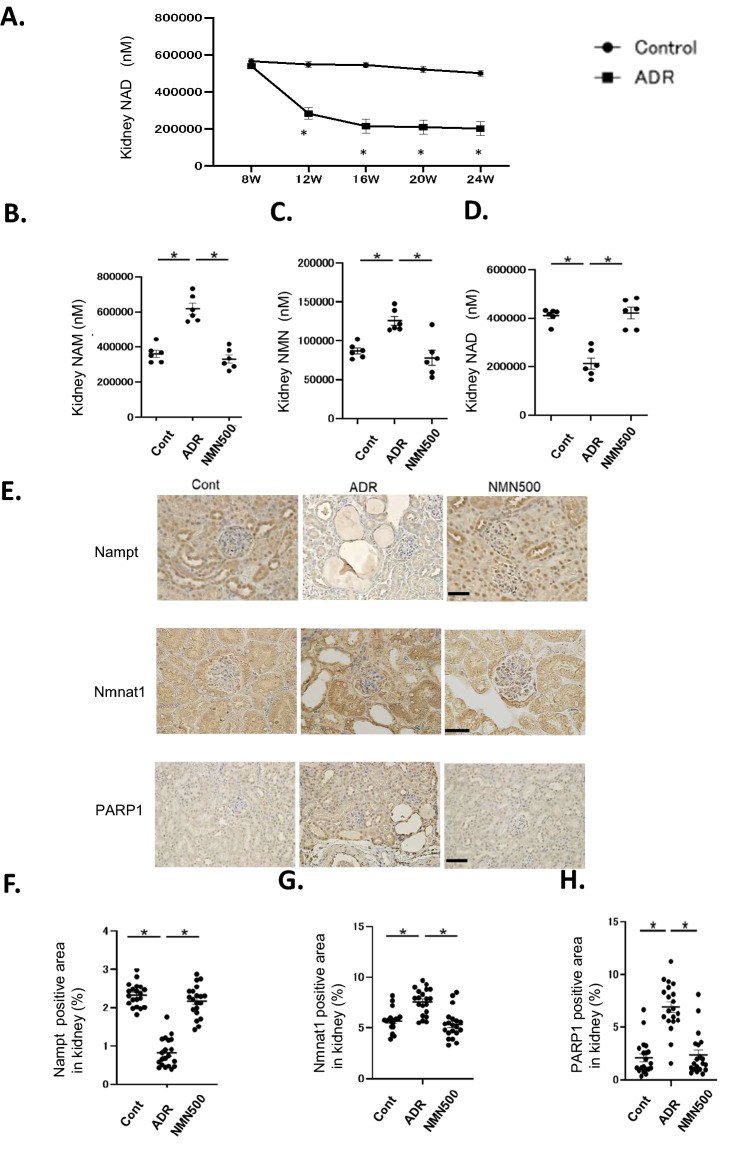
Effects of NMN treatment on NAD^+^ metabolites and the salvage pathway. (**A**) Temporal changes in NAD^+^ concentrations in the kidneys of mice in the Cont and ADR groups (*n* = 6). (**B–D**) Renal tissue concentrations of NAD^+^ metabolites, NAM (**B**), NMN (**C**), and NAD^+^ (**D**) in the salvage pathway on day 28 in the Cont, ADR, and NMN500 groups (*n* = 6). (**E**) Representative images of sections immunostained with Nampt, Nmnat1 in the kidneys of the Cont, ADR, and NMN500 groups (scale bar 50 µm). (**F–H**) Proportional staining areas for Nampt (**F**), Nmnat1 (**G**), and PARP1 (**H**) (*n* = 20 sections/group). All data are shown as mean ± standard error of the mean. Statistical significance between each group is represented by a horizontal bar. *P < 0.05.

The concentrations of NAD^+^ in the kidney were determined from 8 to 24 weeks of age in the Cont and ADR groups to evaluate the chronological changes in NAD^+^ metabolites. The ADR group had lower concentrations of NAD^+^ in the kidneys at 12 weeks of age compared with the Cont group. In the ADR group, the NAD^+^ concentrations in the kidney were further decreased at 16, 20, and 24 weeks of age relative to 8 weeks of age in a time-dependent manner (Fig. 6A). On day 28, the NMN500 group presented with lower concentrations of NAM and NMN in the kidneys compared to the ADR group; no differences in the concentrations of NAM and NMN were observed between the Cont and NMN500 groups (Fig. 6B,C). The concentration of NAD^+^ in the NMN500 group was higher than that in the ADR group (Fig. 6D). Immunohistochemistry revealed that Nampt expression was lower in the ADR group than in the Cont group and higher in the NMN500 group compared with the ADR group (Fig. 6E,F). Conversely, Nmnat1 expression was higher in the ADR group than in the Cont *g*roup, and the upregulation was repressed in the NMN500 group (Fig. 6E,G). The expression of Poly-ADP-ribose-polymerase 1 (PARP1), the major NAD + consumer in cells^25–27^, was increased in the ADR group compared to the Cont group, which might have caused the reduction in NAD^+^ in this group (Fig. 6E,H). Consistent with the changes in NAD^+^ levels (Fig. 6D), PARP1 expression in the kidney was lower in the NMN500 group compared to that in the ADR group (Fig. 6E,H).

### Epigenetic regulatory mechanism of NMN-induced Nmnat1 downregulation

**Figure 7:**
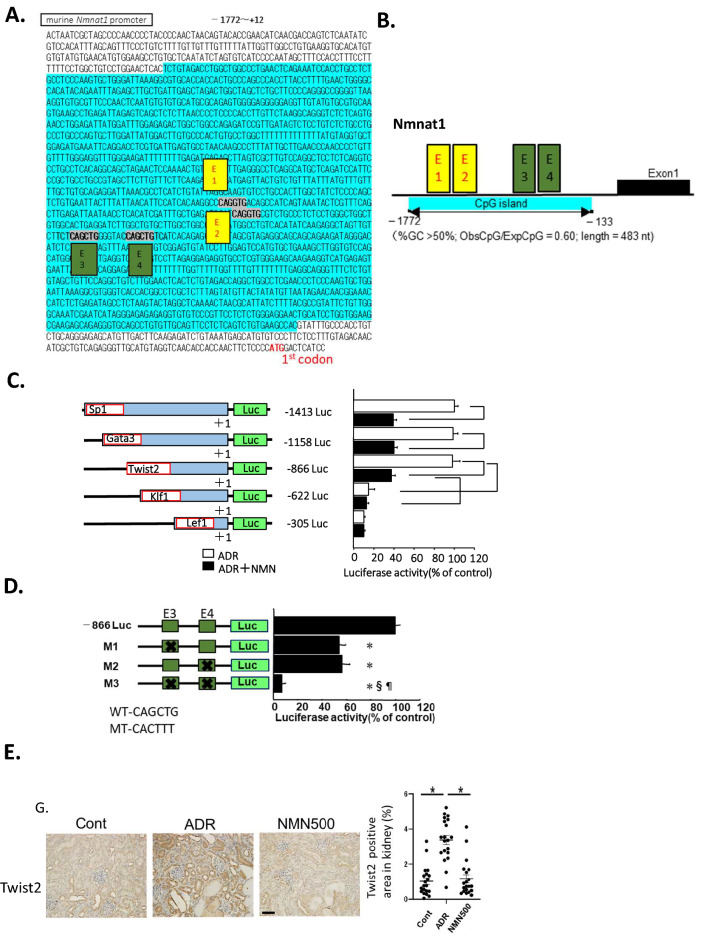
Nmnat1 epigenetic gene regulation by NMN and Twist2. (**A**) Localization and nucleotide sequence in the murine Nmnat1 promoter region. The blue characters represent the putative CpG island mediating effects of ADR or NMN on *Nmnat1* transcription. The transcription start sites are indicated in red. (**B**) Schematic representation of the murine *Nmnat1* gene and promoter. The solid boxes indicate four E-boxes (E1–E4) in the CpG island, highlighted in yellow or green. (**C**) The schematic diagram describes five deletion mutants in the *Nmnat1* promoter sequences (− 1413, − 1158, − 866, − 622, and − 305) that were cloned upstream from a luciferase reporter gene. The bar graphs show the results of transient transfection of the cultured podocytes, illustrating the promoter activities with each deletion. Luciferase activity is shown relative to that of the − 1413 Luc vector in the control vector-transfected cells. Values are expressed as the mean ± the standard error of the mean. *P < 0.05 vs*.* each Luc transfected podocyte (*n* = 3 independent experiments). (**D**) Mutation analysis of *Nmnat1* promoter activity in podocyte cells.* − 866 Luc* WT *Nmnat1*promoter, *M1* distal E3 mutation, *M2* proximal E4 mutation, *M3* mutation in both E3 and E4 corresponding to the Twist2 binding sites. *P < 0.05 vs. –866 Luc in control cells; §P < 0.05 *vs.* –866 Luc in M1 cells; ^¶^*P* < 0.05 vs*.* − 866 Luc in M2 cells (*n* = 3 independent experiments). (**E**) Representative images showing immunostaining for Twist2 in the kidneys of mice from the Cont, ADR, and NMN500 groups (scale bar 50 µm). Proportional staining areas for Twist2 (*n* = 20 sections/group). Statistical significance between each group is represented. *P < 0.05 by ANOVA with Tukey’s posthoc test.

The murine Nmnat1 gene 5′-flanking region (3 kb) was analyzed using the CpGplot program (http://www.ebi.ac.uk/emboss/cpgplot/). A CpG island located in the promoter flanking the first codon was identified (Fig. 7A), and four CAGCTG E-boxes were detected within the island (Fig. 7B). To initially locate the functional regions responsible for regulating Nmnat1 gene expression in podocytes, several 5′ deletion constructs with luciferase as the reporter gene were used for transient transfection studies (Fig. 7C). Sequence analysis using TRANSFAC software revealed the localization of putative transcription factor binding sites for SP-1, Gata3, Twist2, Klf1, and Lef1 within a 1413-bp region in the Nmnat1 promoter (− 1413 to + 1) surrounding the major transcriptional start site (Fig. 7C). Luciferase assays were conducted to measure the ADR-stimulated promoter activities of five deletion constructs (− 1413 Luc, − 1158 Luc, − 866 Luc, − 622 Luc, and − 305 Luc) cloned upstream from luciferase reporter genes in cultured podocytes. The transcriptional activities of the Nmnat1 promoters were not affected in the − 1413 Luc, − 11158 Luc, and − 866 Luc deletion constructs. Following NMN treatment, similar levels of suppression were observed in cells containing these three promoter constructs. Nevertheless, Nmnat1 promoter activity was markedly suppressed in cells transfected with the promoter that deleted the region from − 1808 to − 622, demonstrating similar lowered activities with or without NMN. These results implied that the promoter region spanning − 866 to − 622 is essential for ADR-induced Nmnat1expression and the NMN-induced suppression of Nmnat1 gene expression. The TRANSFAC analysis showed that this ADR or NMN response region (between − 866 and − 622) contained consensus sites for Twist2 binding (Fig. 7C), corresponding to the Enhancer Box (E-box) sites. It indicates that Twist2 was the principal DNA-binding component of this protein–DNA complex. Luciferase assays conducted with the mutated Twist2 consensus sites (one or both) showed that both sites were functional (Fig. 7D). The E-box sites were located within the surrounding CG-rich sequences. Additionally, a computer search indicated that the CpG islands, the well-known targets of epigenetic modifications, resided within the Nmnat1 gene (Fig. 7A). Thus, the regulation of Nmnat1 expression by NMN appeared to be influenced by the epigenetic mechanisms of DNA methylation. IHC using Twist2 was conducted on day 28 (Fig. 7E); the expression of Twist2 was increased in the ADR group than in the Cont group but suppressed in the NMN500 group.

### Dnmt1 repressed Twist2 binding activity in the Nmnat1 E-box

**Figure 8:**
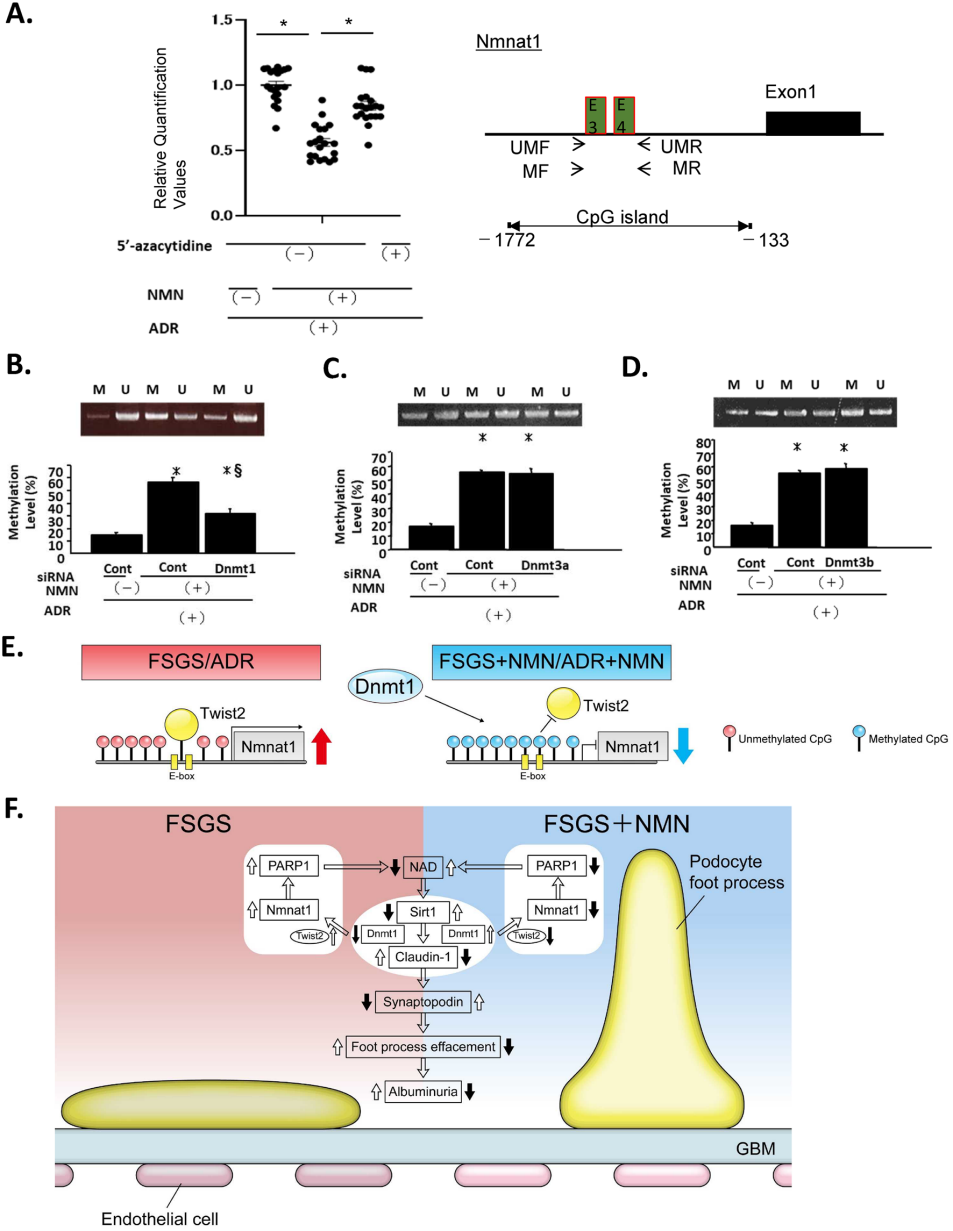
Nmnat1 epigenetic gene regulation by NMN and Dnmt1. (**A**) Promoter methylation downregulated *Nmnat1* gene expression after NMN administration. The cells were treated with 5′-azacytidine (1 µM) for 4 days before incubation with NMN (100 µM) for 24 h. The *Nmnat1* gene expression level was quantified by real-time polymerase chain reaction (PCR); *n* = 3 independent experiments. The positions of primers used for MSP. *UMF* unmethylated forward primer, *UMR* unmethylated reverse primer, *MF* methylated forward primer, *MR* methylated reverse primer. (**B–D**) The promoter methylation levels were examined by methylation-specific PCR (MSP). Methylation of the *Nmnat1* promoter with or without NMN and a siRNA for *Dnmt1 *(**B**), *Dnmt3a* (**C**), and *Dnmt3b* (**D**). The upper panels show representative bands of MSP and the lower panels show the results of real-time MSP (*n* = 3 independent experiments). (**E**) Schema depicting the epigenetic regulation of the expression of *Nmnat1* by Dnmt1. In the FSGS state or after adriamycin treatment, the methylation level of the *Nmnat1* promoter region was low. Thus, Twist2 could bind to the E-box sites and maintain the high expression level of *Nmnat1*. In the presence of NMN, the methylation in this region is increased by Dnmt1. Consequently, the expression level of *Nmnat1* is decreased because Twist2 cannot bind to the E-box sites. Statistical significance between each group is represented. *P < 0.05 by ANOVA with Tukey’s post hoc test. (**F**) Schematic model of NMN action in ADR-induced FSGS. In this study, we investigated the effect of a preemptive short-term NMN treatment on ADR-induced FSGS. This transient treatment reduced albuminuria immediately after treatment until 2 weeks after the treatment. We further demonstrated that NMN treatment retained the levels of NAD^+^ in the kidney by suppressing the NMN consumer Nmnat1 and the NAD consumer PARP1 in the NAD^+^ salvage pathway. Furthermore, NMN treatment increased Sirt1 expression and downregulated Claudin-1 expression, leading to the attenuation of the downregulation of Synaptopodin and the effacement of the podocyte foot processes. Therefore, this method could be a preventive strategy against FSGS.

Pretreatment of podocytes with 5′-azacytidine followed by incubation with NMN lysate led to a significant recovery of Nmnat1 gene expression (Fig. 8A). These data suggested that DNA methylation induced by NMN prevented Twist2 from binding to the E-box sites. To confirm the involvement of DNA methyltransferase (Dnmt) in the methylation of CpG sites in the Nmnat1 gene, the podocytes were transfected with siRNA for Dnmt1, Dnmt3a, or Dnmt3b. Methylation was significantly increased in cells treated with NMN when compared with those without NMN treatment. The methylation with NMN was suppressed by a siRNA for Dnmt1 but not for Dnmt3a or Dnmt3b (Fig. 8B–D). Taken together, these findings indicated that Nmnat1 gene expression can be regulated epigenetically through CpG methylation by Dnmt1, which was recruited following the incubation of cells with NMN. The methylation of the Nmnat1 promoter region was low following treatment with ADR; thus Twist2 could bind to the E-box sites and maintain the high expression level of *Nmnat1*. In the presence of NMN, the methylation levels in the E-box were elevated by the recruited DNMT1; consequently, Twist2 could not bind to the E-box sites, which resulted in a decreased expression of *Nmnat1* (Fig. 8E,F).

### Sirt1, Sirt3, Sirt6, and Nmnat1 expression in human renal biopsy specimens of FSGS and IgA nephropathy

Intra-renal expression levels of Sirt1, Sirt3, Sirt6, and Nmnat1 were evaluated in specimens from 27 patients who were histologically diagnosed with FSGS (Supplementary Table 1, Supplementary Fig. 1) and IgA nephropathy (Supplementary Table 2, Supplementary Fig. 2). Sirt1 and Sirt6 expression levels were decreased, whereas Nmnat1 expression was increased, in patients with heavy proteinuria when compared to FSGS patients with low levels of proteinuria (Supplementary Fig. 1). Sirt3 levels were unchanged between FSGS patients with high vs low proteinuria. Among clinical parameters, proteinuria was positively correlated with Nnamt1 expression and negatively correlated with Sirt1 and Sirt6 expression. No correlations were observed between Sirt1 or Sirt6 expression and eGFR. We also examined intra-renal expression levels of Sirt1, Sirt3, Sirt6, and Nmnat1 in specimens from patients with IgA nephropathy (Supplementary Fig. 2). No differences in immunostaining intensities for Sirt1, Sirt3, Sirt6, or Nmnat1 were observed between samples from patients with heavy proteinuria and low proteinuria. Accordingly, there were no correlations between the expression levels of these proteins and proteinuria. Moreover, no correlations were observed between immunostaining intensities for Sirt1, Sirt3, Sirt6, or Nmnat1 and eGFR.

### NMN attenuates renal tubulointerstitial damage

Tubulointerstitial lesion index scoring of kidney sections confirmed that ADR mice had the greatest degree of tubulointerstitial damage. NMN treatment led to a marked reduction in tubulointerstitial lesion index scores (Supplementary Fig. 3A). qRT-PCR was performed to measure mRNA expression levels of Kim-1, cubilin, and αSMA. A trend toward lower renal cubilin mRNA levels was observed in the ADR group compared with the control group. Cubulin mRNA were significant higher in the ADR + NMN group (Supplementary Fig. 3B). Kim-1 and αSMA mRNA levels were increased in the ADR mice compared with the control group, and treatment with NMN ameliorated this increase (Supplementary Fig. 3B). These data are consistent with the known anti-fibrotic effects of NMN treatment.

### Survival rate curves

Murine survival rates in the present study were evaluated by Kaplan–Meier and log rank tests, which revealed that short-term treatment with NMN was associated with increased survival time (Supplementary Fig. 4). NMN treatment was associated with 0.20-fold lower death rates (*P* = 0.045) in male *ADR* mice.

## Discussion

In the present study, the administration of NMN ameliorated kidney damage, both functionally and histologically, in the murine ADR-induced nephropathy model. NMN maintained the NAD^+^ levels in the kidneys of the ADR-treated mice and altered the expression levels of Sirt1, Nampt, and Nmnat1. These changes were evident and sustained even after the discontinuation of the short-term NMN treatment, thus indicating that the effects of this treatment protocol were continuous. This study provides a proof of concept for the transient short-term administration of NMN as an effective treatment for proteinuric renal disease in the FSGS model.

Fourteen days of NMN treatment led to a persistent reduction in albuminuria in FSGS and an amelioration in histological changes such as foot process effacement and glomerular sclerosis. We have previously shown that decreased Sirt1 in podocytes epigenetically upregulated the level of Claudin-1 (through Dnmt1 activation) and reduced the level of Synaptopodin, subsequently causing foot process effacement and albuminuria. Consistent with the results of our previous study, IHC demonstrated low Sirt1, high Claudin-1, and low Synaptopodin expression levels in the glomeruli of the ADR mice in the present study. Furthermore, the expression levels of H3K9me2 and DNMT1 were lower in the glomeruli of the ADR mice. These unfavorable changes were ameliorated by NMN, even after the termination of the treatment, thereby suggesting that Sirt1 reactivation halts the aggravation of the molecular changes in Sirt1-Claudin-1-Synaptopodin via its long-lasting epigenetic effects.

Chronologically decreased NAD^+^ concentrations were observed in the ADR group. Surprisingly, the NAD^+^ levels in the kidneys of the NMN-treated ADR mice were higher on day 28, corresponding to those at 2 weeks after the termination of the treatment period. NMN is rapidly converted to NAD^+^ and disappears from blood and the organs within 15 min; furthermore, the half-life of NAD is less than 10 h^28–30^. Paradoxically, low levels of NMN were observed on day 28 in the NMN500 group, despite prior supplementation. These findings indicate that the short-term treatment modified the salvage pathway for a long period. NMN treatment upregulated Nampt expression and downregulated Nmnat1 expression on day 28. It can be surmised that NMN treatment suppresses the overconsumption of NMN by repressing the NMN consumer, Nmnat1 (Fig. 6E,G). Moreover, Nmnat1 was shown to directly bind to and activate PARP1^26^. In another study, Nmnat1 was shown to not only synthesize NAD^+^ but also stimulate PARP1 activity independently of NAD^+^ synthesis^27^. Taken together, these findings indicated that NMN treatment blocked the overconsumption of NMN and NAD, which was evoked by podocyte damage caused by ADR.

Some studies have reported that lower doses of NMN can improve the pathogenesis to a greater degree than higher doses^31^. In one study, marked improvements in oxygen consumption, energy expenditure, and physical activity were observed with 100 mg/kg of NMN when compared with those with 300 mg/kg NMN^31^. In another report, reduced cell death in the CA1 neurons was best achieved with 62.5 mg/kg of NMN^32^. Conversely, one study reported the dose-dependent effects of NMN treatment on body weight, bone density, and some age-related changes^31^. The dose–response experiment in the present study demonstrated that NMN treatment using dosages of 300 and 500 mg/kg improved albuminuria, whereas a dosage of 100 mg/kg had no effect. Hence, the ideal dosage might vary depending on the organs involved and the pathogenesis. In terms of the adverse, 1 year of treatment with 100 and 300 mg/kg/day of NMN orally appeared to be tolerable by the patients^31^. One study reported that 90 days of treatment with 3000 mg/kg of NR resulted in several adverse metabolic and histological effects, including an increase in kidney weight and the presence of basophilic tubules, tubular atrophy, focal segmental glomerulosclerosis, and monocyte infiltration in the kidneys; nevertheless, treatment with 300 mg/kg of NR for 90 days had no adverse effects^33^. In the present study, the short-term transient treatment was adopted for 2 weeks. Nonetheless, no obvious adverse effects were observed, thus supporting the feasibility of this treatment protocol.

Twist2 (dermo-1) is a basic helix-loop-helix (bHLH) transcription factor, which recognizes the E-box^34^. Twist2 has 66% identical homology and an overlapping pattern of cellular expression with the more studied Twist1^35^. The role of Twist1 in renal pathophysiology is emerging^36–38^. However, the detailed role of Twist2 in the kidneys has not been fully elucidated. Importantly, we identified a pathological transcription factor, Twist2, which might mediate the FSGS-dependent increase in glomerular sclerosis; additionally, we showed that a reduction in Twist2 expression by NMN is a potential intervention that can attenuate progressive FSGS. The inactivation of Twist2 by an endogenous ligand or exogenous substance like NMN might regulate the expression of *Nmnat1* and reduce glomerular sclerosis due to PARP1 suppression. Glomerular sclerosis is known as a pivotal pathway not only in FSGS but also in other kidney diseases leading to the progression of CKD. Hence, further studies are required to evaluate whether a therapeutic strategy that compensates for the downregulation of *Nmnat1*, such as NMN administration, would prove effective in protecting against the progression of CKD.

In the present study, NMN decreased the level of PARP1, which is directly regulated by Nmnat1. Nmnat1 protein expression via epigenetic regulation of increasing methylation of the *Nmnat1* promoter region, an effect mediated by Dnmt1. Furthermore, this study demonstrated that Dnmt1 induces the methylation of the binding site in Twist2, and the subsequent reduction in its binding to the Nmnat1 promoter is the initial step for transcriptional regulation. Additional studies are required to elucidate that Nmnat1 downregulation can attenuate glomerular sclerosis. Details regarding the mechanism by which renal Dnmt1 is activated remain unknown, but reports suggest that the expression and/or activity of Dnmt1 can be increased via Sirt1 activation^39,40^.

Nampt deficiency leads to the inactivation of Sirtuin^6^. Significant expression levels of Sirt1, Sirt3, and Sirt6 were observed in normal kidneys. Among these isoforms, the ADR-treated mice exhibited a significantly decreased expression of Sirt1 and Sirt6, whereas that of Sirt3 was unaltered. NMN rescued the expression levels of Sirt1 and Sirt6 but did not affect that of Sirt3. These differences in the isoforms of Sirtuin might be associated with differences in the cellular fraction; nonetheless, further investigations are needed. Several studies, including the current study, demonstrated that Sirt6 deficiency induces podocyte damage^41^ and renal fibrosis^6^. Thus, the decrease in Sirt6 expression, besides the Sirt1-related pathway, might have led to renal damage in the ADR mice in the present study.

We measured NAD levels using whole kidney samples for each group. NAD is readily dissolved as it is a small nucleotide molecule. Accordingly, the measurement of segmental NAD levels from tissue samples is technically challenging. However, future technical advances allowing accurate measurement of NAD levels from specific renal compartments may elucidate the effect of tubular NAD levels on glomerular NAD levels and, vice versa, the effect of glomerular NAD levels on tubular NAD levels.

Nampt expression levels were reduced in ADR-treated FSGS models and normalized in response to NMN treatment. This pattern of expression was similar to that of Sirt1. Sirt1 levels were also decreased in ADR-treated FSGS models, with levels seen to normalize in response to NMN treatment. Previous reports have demonstrated that Sirt1 directly upregulates Nampt promoter activity and its concomitant protein level expression via a positive feedback loop mediated by Sirt1-deacetylated transcripiton factors such as cMyc^42^. Other reports have indicated that Sirt1 directly deacetylates Nampt, which stabilizes Nampt protein via post-translational mechanisms^43^. Taken together, these findings indicate NMN treatment increases both Sirt1 and Nampt levels via a positive feedback mechanism.

Young’s glomerular module has utility in measuring glomerular elasticity and stiffness^44,45^; however, this method is technically challenging. A limitation of the present study was the lack of glomerular elasticity measurements. Further studies are required to determine the effects of ADR and NMN on glomerular stiffness and elasticity.

In conclusion, supplementation with short-term NMN for two weeks sufficiently restored and maintained NAD^+^ and Sirt1 levels and protected the kidneys from FSGS 2 weeks after the termination of the treatment in mice. This study provides evidence of the long-term effects of NMN treatment; additionally, it demonstrates that short-term NMN supplementation is sufficient to suppress the progression of FSGS.

## Methods

### Animal experiment protocols

Male BALB/c mice (8 weeks old) were purchased from Japan CLEA Co. (Tokyo, Japan). They were housed at a constant room temperature of 22  ± 1 °C under a controlled 12 h light/12 h dark cycle and had free access to water and regular chow. The mice were intravenously injected with a vehicle (normal saline; n = 12) or 11 mg/kg of ADR (n = 24) Santa Cruz Biotechnology Inc., Dallas, TX, USA) on day 0^23^. Twenty mice treated with normal saline were assigned to the non-FSGS control (Cont) group. The ADR-treated mice were randomly assigned to two groups (12 per group) as follows: those treated with vehicle (normal saline; ADR group) and those treated with NMN (500 mg/kg/day) in normal saline (NMN 500 group). The animals were treated every day for 14 consecutive days from day 0 to day 14, as described previously^22^. The survival of the animals was examined every day, and the body weights were estimated every week. Urine samples were collected on days 14 and 28. Serum samples measuring the cholesterol and creatinine levels were collected on days 14 and 28. The kidneys of the animals were harvested to assess the renal histology on day 14 (just after completing the NMN treatment) and day 28 (2 weeks after treatment termination). All the animal studies were approved by the Animal Care Committee and the Ethics Committe of the Tokushima University School of Medicine and study was carried out according to the national and regional guidelines. All the studies are reported in accordance with ARRIVE guidelines. All surgeries were performed afer intraperitoneal injection of 0.3 mg/kg medetomidine, 4.0 mg/kg midazolam, and 5.0 mg/kg butorphanol, and all eforts were made to minimize animal sufering.

### Blood and urine examination

Urine was collected for 24 h from metabolic cages, and the renal function was evaluated based on the serum creatinine levels and creatinine clearance (Ccr). The Ccr was calculated using the following formula: urinary creatinine × urine volume/serum creatinine/1440, where 1440 represents the number of minutes in 24 h. Albuminuria was assessed based on the urine albumin to creatinine ratio (ACR). The urine albumin level was assessed by an enzyme-linked immunosorbent assay (ELISA; Albuwell M; Ethos Biosciences, Pennsylvania, USA). The urine and serum creatinine levels were assessed using the QuantiChrom™ Creatinine Assay Kit (BioAssay Systems, California, USA). Serum cholesterol levels were measured with a mouse cholesterol ELISA Kit (Abcam, ab285242).

### Histology and immunohistochemistry of the kidney

Images from at least 20 sequential glomerular cross-sections divided approximately at the glomerular equator were collected for each histological section by blinded observers. PAS-stained samples from 20 consecutive glomeruli per animal were examined. The glomerular surface area was traced along the outline of the capillary loop using Image-Pro Plus 7.0J software (Media Cybernetics, Silver Spring, MD, USA). For the quantitative analysis of the mesangial expansion, the PAS-positive area in the glomeruli was evaluated. Specifically, a minimum hue–saturation–intensity threshold was set on Image-Pro Plus 7.0J (Media Cybernetics), and the area exceeding this threshold was counted as a PAS-positive area. Consequently, the percentage of PAS-positive area per glomeruli was calculated. IHC was performed as described previously^3^. Briefly, paraffin sections (4 µm) were fixed in 3% formaldehyde and stained with the primary antibodies for Claudin-1 (Invitrogen, 51-9000, 1:50), Sirt1 (Sigma-Aldrich, 07-131, 1:100), Synaptopodin (Fitzgerald, 10R-S125A, undiluted), WT-1 (Santa Cruz, C-19, 1:200), Nampt (Bethyl Laboratories, A300-372A, 1:500), Nmnat1 (Proteintech, 11399-1AP, 1:500), Sirt3 (Cell Signaling, C73E3, 1:50), Sirt6 (LSBio, aa250-334, 1:2500), DNMT1 (Cell Signaling Technology, #5032, 1:100), PARP1 (Proteintech, 13371-1-AP, 1:200), Twist2 (Abcam, ab66031, 1:200), and H3K9me2 (Abcam; mAbcam 1220, 1:200). Goat antirabbit IgG (Nichirei, 414341) and goat antimouse IgG (Nichirei, 414321) antibodies were used as the secondary antibodies. All sections were examined under a light microscope (Olympus BX53 microscope) and digitized with a high-resolution camera. For the quantitative analysis of the staining for Sirt1, Claudin-1, Synaptopodin, Sirt3, Sirt6, H3K9me2, and DNMT1, the DAB-stained area per glomerular surface area was calculated using Image-Pro Plus 7.0J. The Definiens Tissue Studio software (Definiens, Munich, Germany) was used to calculate the DAB-stained area per section per kidney for the quantitative analysis of the Nampt, Nmnat1, PARP1, and Twist2 immunostaining. All assessments were performed in a blinded manner, and four kidneys were examined in each group.

### Electron microscopy

For the electron microscopy (EM) evaluation, the kidney tissues were harvested and fixed overnight at 4 °C with 2% paraformaldehyde and 2% glutaraldehyde (GA) in 0.1 M phosphate buffer (PB; pH 7.4). After fixation, the samples were washed three times with 0.1 M PB for 30 min each and post-fixed with 2% osmium tetroxide (OsO4) in 0.1 M PB at 4 °C for 2 h. The fixed tissue blocks were embedded in Epon epoxy resin. The average number of podocyte foot processes was counted and divided by the glomerular basement membrane (GBM) length (µm) to determine the densities of the foot processes as described previously^22^. The counts were performed on 105 micrographs from at least three glomeruli in each mouse. Using Image-Pro Plus 7.0J, the length and thickness of the GBM were measured.

### NAD^+^ metabolite measurement

Levels of NAD^+^ metabolites were measured using LC/MS/MS as described previously^3^ with minor modifications. Briefly, three volumes of methanol containing 6% perchloric acid and 4% phosphoric acid were used to homogenize the tissues. Subsequently, three volumes of methanol (including the deuterated internal standard) were added to the tissue homogenate or in serum samples; this mixture was vortexed and centrifuged. The supernatant was diluted with water and LC/MS/MS was used to analyze it. The Shimadzu Nexera UHPLC system (Shimadzu, Kyoto, Japan)—consisting of an LC-30 AD pump, a DGU-20A5R degasser, a CTO-20AC column oven, and a SIL-30ACMP autosampler—was used. At 50 °C, separation was carried out using a Triart C18 column (3.0 150 mm, 5 m, YMC, Kyoto, Japan). Mobile phase A included water/formic acid/undecafluorohexanoic acid (1000/0.1/0.2, v/v/v), and mobile phase B included methanol. The chromatographic conditions were 0–4 min (5%–80% B, 0.5 mL/min), 4–4.01 min (80%–95% B, 0.5–1.0 mL/min), 4.01–7 min (95% B, 1.0 mL/min), 7–7.01 min (95%–5% B, 1.0–0.5 mL/min), and 7.01–13 min (5% B, 0.5 mL/min). An API5000 triple quadrupole mass spectrometer (SCIEX, Framingham, MA, USA) with electrospray ionization (ESI) in the positive ion mode was used for mass spectrometric detection. Standard solutions were used to optimize the ESI–MS/MS settings for each analyte. Quantitation was performed using multiple reaction monitoring with the following transitions: m/z 123 → 80 for NAM, m/z 335 → 123 for NMN, and m/z 664 → 136 for NAD^+^.

### *Nmnat1* CpG methylation in vitro by methylation-specific polymerase chain reaction (MSP) and real-time MSP

Total genomic DNA from cultured podocytes was extracted using the DNeasy Kit (Qiagen Japan, Tokyo, Japan). Bisulfite conversion of genomic DNA was performed using a Zymo EZ DNA Methylation Gold kit (Zymo Research Corp., Orange, CA, USA). MSP was performed to determine the methylation status of the *Nmnat1* gene and real-time MSP was performed to quantitatively analyze the methylation of the gene, as described previously^24^. Supplementary Table 3 lists the specific methylated or unmethylated sequences of the primer sets. Three independent MSPs and real-time MSPs were performed.

### Luciferase assay

A 1414-bp fragment (− 1413 to + 1) of the 5′ flanking region of *Nmnat1* was isolated from the murine BAC genomic clone using the restriction endonucleases *Bal*I and *EcoT14I*. Plasmids − 1158, − 866, − 622, and − 305 Luc were prepared by subcloning the *BglI*, *ClaI*, *HindIII*, and *Sca*I inserts from − 1413 Luc. These Nmnat1/pGL3 plasmids (− 1413 Luc, − 1158 Luc, − 866 Luc, − 622 Luc, and − 305 Luc) containing the murine Nmnat1 promoter sequences between − 1413, − 1158, − 866, − 622, and − 305 and + 1 were fused to a pGL3 vector, a firefly luciferase reporter plasmid, and then transfected with Lipofectamine 2000 (Invitrogen). NMN and ADR were added and pRL-CMV (Renilla luciferase reporter vector; Promega, Madison, WI, USA) was cotransfected into the cells. Murine podocyte cells have been described previously^5^. Podocyte cells were treated with 0.2 μg/ml of ADR in a regular medium, and the medium was harvested at 24 h after treatment. The luciferase activity was measured as described previously^24^. The mutagenesis primers were generated from the − 866 luciferase reporter plasmid by mutating CAGCTGA to CCCTTTA using in vitro mutagenesis.

### Culture of podocytes

Conditionally immortalized mouse podocytes were donated by P. Mundel (Mt. Sinai School of Medicine, New York, NY, USA) and K. Asanuma (Chiba University, Chiba, Japan). The podocytes were seeded at a density of 5 × 10^5^ per 100 mm^2^, incubated for 7 days (differentiation), and used for further experiments. The differentiation of the cells was confirmed as described previously^5^. The cells were treated with 5 μM 5-aza-dC (Sigma-Aldrich) for 96 h. For the *Dnmt* siRNA treatment, a *Dnmt* siRNA duplex was purchased from Sigma-Aldrich. The sense sequences were 5′-[dT] GGAAUGGCAGAUGCCAACAGC [dT]-3′ for Dnmt1, 5′-[dT] GAAAGCGAAGGUCAUUGCA [dT]-3′ for Dnmt3a and 5′-[dT] GCUAGCGAAGGUCAUUGCA [dT]-3′ for Dnmt3b. The control siRNA consisted of a scrambled siRNA construct encoding a nonspecific siRNA without mammalian homology. These siRNAs (100 pmol μl^−1^) were transfected using Lipofectamine 2000 (Invitrogen) for 24 h.

### Tubulointerstitial lesion index scoring

Under anesthesia, kidneys were removed and fixed with 4% paraformaldehyde. Fixed kidneys were subsequently embedded in paraffin and 4 µm sections were cut and stained with periodic acid-Schiff (PAS). Tubulointerstitial injury (defined as tubular atrophy, dilatation, thickening of the basement membrane, or protein casts) was assessed by semi-quantitative analysis^46,47^. Twenty cortical fields from each animal were examined and graded according to a scale of 0–4 as follows: 0, no tubulointerstitial injury; 1, 25% of the tubulointerstitium injured; 2, 25%–50% of the tubulointerstitium injured; 3, 51%–75% of the tubulointerstitium injured; and 4, 76%–100% of the tubulointerstitium injured. All sections were examined in a blind manner.

### Quantitative RT-PCR

Total RNA was extracted from murine renal cortex samples and cultured cells using RNAiso Plus (Takara, Japan) according to the manufacturer’s instructions. Reverse transcription and quantitative renal-time PCR were performed using PrimeScript RT reagent kits and SYBR Premix Ex Taq (Takara, Japan). All data are reported as the mean ± standard error of the mean (S.E.M) normalized to GAPDH. Primer sequences used in the present study were as follows: GAPDH sense: 5′-GTC TTCACTACCATGGAGAAGG-3′ and antisense: 5′-TCATGGATGACCTTGGCC; α-smooth muscle actin (αSMA) sense: 5′-CCCTGAAGAGCATCC GACA-3′ and antisense: 5′-CCAGAGTCCAGCACAATACC-3′; Kim-1 sense: 5′-TCAGAAGAGCAGTCGGTACAAC-3′ amd antisense: 5′-TGTAGCTGTGGGCCTTGTAG-3′; Cubilin sense: 5′-AGCTCAACCTCCATTCAATCATA-3′ and antisense: 5′-GTGCAATCTGTGCTGCTT-3′.

### Animal survival analysis

The mice were evaluated for the survival analysis. A Kaplan–Meyer survival analysis and Log rank test were performed. *P* < 0.05 was considered statistically significant.

### Human renal specimens from needle biopsy

Needle renal biopsy specimens were obtained from 27 patients with FSGS and 17 patients with IgA nephropathy. The present study was performed according to the declaration of Helsinki, and the study protocol was approved by the Human Ethics Review Committee of the Tokyo Dental College Ichikawa General Hospital and the Tokushima University School of Medicine.

### Statistical analyses

GraphPad Prism 8 software (GraphPad Software, CA, USA) was used to perform the statistical analyses. Data are expressed as means ± standard error of the mean. Comparisons among several groups were analyzed using a one-way analysis of variance and Tukey’s post hoc test. A *P *value of < 0.05 was considered statistically significant.

## Supplementary Information

Supplementary Information.

## Data Availability

The data that support the findings of this study are avail-able in the methods of this article. Further information and requests for resources and reagents are available from the corresponding author (kazuhiro@tokushima-u.ac.jp).
