# Supplementary material for: Nicotinamide mononucleotide ameliorates adriamycin-induced renal damage by epigenetically suppressing the NMN/NAD consumers mediated by Twist2
Source: Sci Rep. 2022 Aug 12;12:13712. doi: 10.1038/s41598-022-18147-2 (PMC9374671; doi:10.1038/s41598-022-18147-2)
Supplement: Supplementary file 1 — Supplementary Information. [file 41598_2022_18147_MOESM1_ESM.pdf]

# Clinilcal data of patients with FSGS.

Clinical parameters in patients with FSGS at the time of needle renal biopsy.

| Sample name | Age (years) | Gender | Serum creatinine (mg/dl) | eGFR (ml/min/1.73m$^2$) | Proteinuria （g /day) | Total serum protein (g/dl) |
|---|---|---|---|---|---|---|
| FSGS-1 | 26 | Male | 2.08 | 34.2 | 1.90 | 6.7 |
| FSGS-2 | 55 | Male | 2.52 | 22.3 | 5.70 | 6.8 |
| FSGS-3 | 72 | Female | 2.89 | 13.2 | 3.50 | 6.1 |
| FSGS-4 | 63 | Male | 1.93 | 28.8 | 3.80 | 5.6 |
| FSGS-5 | 55 | Male | 2.13 | 26.9 | 4.90 | 6.4 |
| FSGS-6 | 96 | Male | 3.31 | 14.1 | 2.10 | 4.9 |
| FSGS-7 | 75 | Male | 4.31 | 11.4 | 0.30 | 5.9 |
| FSGS-8 | 63 | Male | 1.86 | 30.0 | 4.90 | 6.6 |
| FSGS-9 | 59 | Female | 1.48 | 29.0 | 1.60 | 6.8 |
| FSGS-10 | 51 | Male | 0.80 | 80.1 | 2.60 | 6.7 |
| FSGS-11 | 27 | Female | 0.79 | 97.5 | 0.79 | 7.2 |
| FSGS-12 | 86 | Male | 1.51 | 25.4 | 3.50 | 6.5 |
| FSGS-13 | 41 | Female | 0.67 | 76.5 | 1.40 | 7.2 |
| FSGS-14 | 78 | Male | 9.47 | 4.7 | 0.31 | 4.7 |
| FSGS-15 | 80 | Male | 2.55 | 19.8 | 0.53 | 6.5 |
| FSGS-16 | 83 | Male | 1.21 | 44.3 | 8.39 | 4.8 |
| FSGS-17 | 23 | Male | 0.75 | 108.1 | 1.16 | 5.1 |
| FSGS-18 | 78 | Male | 1.29 | 42.1 | 7.26 | 6.2 |
| FSGS-19 | 44 | Male | 0.72 | 93.8 | 2.27 | 6.1 |
| FSGS-20 | 67 | Male | 1.35 | 41.8 | 3.49 | 6.8 |
| FSGS-21 | 27 | Male | 2.54 | 27.2 | 4.36 | 6.0 |
| FSGS-22 | 74 | Male | 3.24 | 15.6 | 0.16 | 7.4 |
| FSGS-23 | 81 | Female | 2.56 | 14.5 | 0.30 | 7.5 |
| FSGS-24 | 83 | Male | 4.20 | 11.4 | 3.33 | 6.4 |
| FSGS-25 | 58 | Male | 10.76 | 4.5 | 15.59 | 4.6 |
| FSGS-26 | 23 | Male | 1.15 | 67.7 | 2.54 | 7.1 |
| FSGS-27 | 33 | Female | 0.74 | 73.1 | 3.17 | 6.7 |

Supplementary Table 1 , Hasegawa *et al.*

# Clinilcal data of patients with IgA nephropathy.

Clinical parameters in patients with IgA nephropathy at the time of needle renal biopsy.

| Sample name | Age (years) | Gender | Serum creatinine (mg/dl) | eGFR (ml/min/1.73m$^2$) | Proteinuria （g /day) | Total serum protein (g/dl) |
|---|---|---|---|---|---|---|
| IgA-1 | 49 | F | 0.87 | 55 | 0.79 | 6.5 |
| IgA-2 | 16 | F | 0.70 | 96 | 0.68 | 7.1 |
| IgA-3 | 21 | F | 0.57 | 110 | 0.95 | 7.3 |
| IgA-4 | 25 | F | 0.68 | 87 | 1.12 | 6.8 |
| IgA-5 | 32 | M | 1.06 | 67 | 1.41 | 6.9 |
| IgA-6 | 69 | F | 0.68 | 64 | 2.55 | 6.7 |
| IgA-7 | 43 | F | 1.09 | 44 | 1.92 | 7.2 |
| IgA-8 | 45 | F | 0.68 | 73 | 1.18 | 6.3 |
| IgA-9 | 45 | M | 1.18 | 54 | 1.21 | 6.4 |
| IgA-10 | 19 | F | 0.69 | 92 | 1.05 | 7.1 |
| IgA-11 | 55 | F | 0.91 | 50 | 2.32 | 6.8 |
| IgA-12 | 51 | F | 0.57 | 86 | 1.47 | 7.0 |
| IgA-13 | 38 | F | 0.77 | 67 | 1.02 | 6.9 |
| IgA-14 | 38 | F | 0.75 | 69 | 2.79 | 6.7 |
| IgA-15 | 51 | F | 0.68 | 71 | 1.55 | 6.6 |
| IgA-16 | 37 | M | 1.28 | 53 | 1.45 | 6.9 |
| IgA-17 | 46 | M | 1.36 | 46 | 3.8 | 6.3 |

# MSP primers for analysis of Nmnat1 gene methylation.

UF and UR indicate the primers specific to unmethylated targets. MF and MR indicated the primers specific to methylated targets.
F, forward; R, reverse; MSP, methylation-specific PCR.

| Gene name | Primers | Sequence (5′-3′) |
|---|---|---|
| *Nmnat1* | MF | TTTTGGTTGTGTTTGGTTGG |
| | MR | AAAAACCCGACACCTCAACA |
| | UF | TTTTGGTTGTGTTTGGTTGG |
| | UR | AAAAACCAGACACCTCAACA |

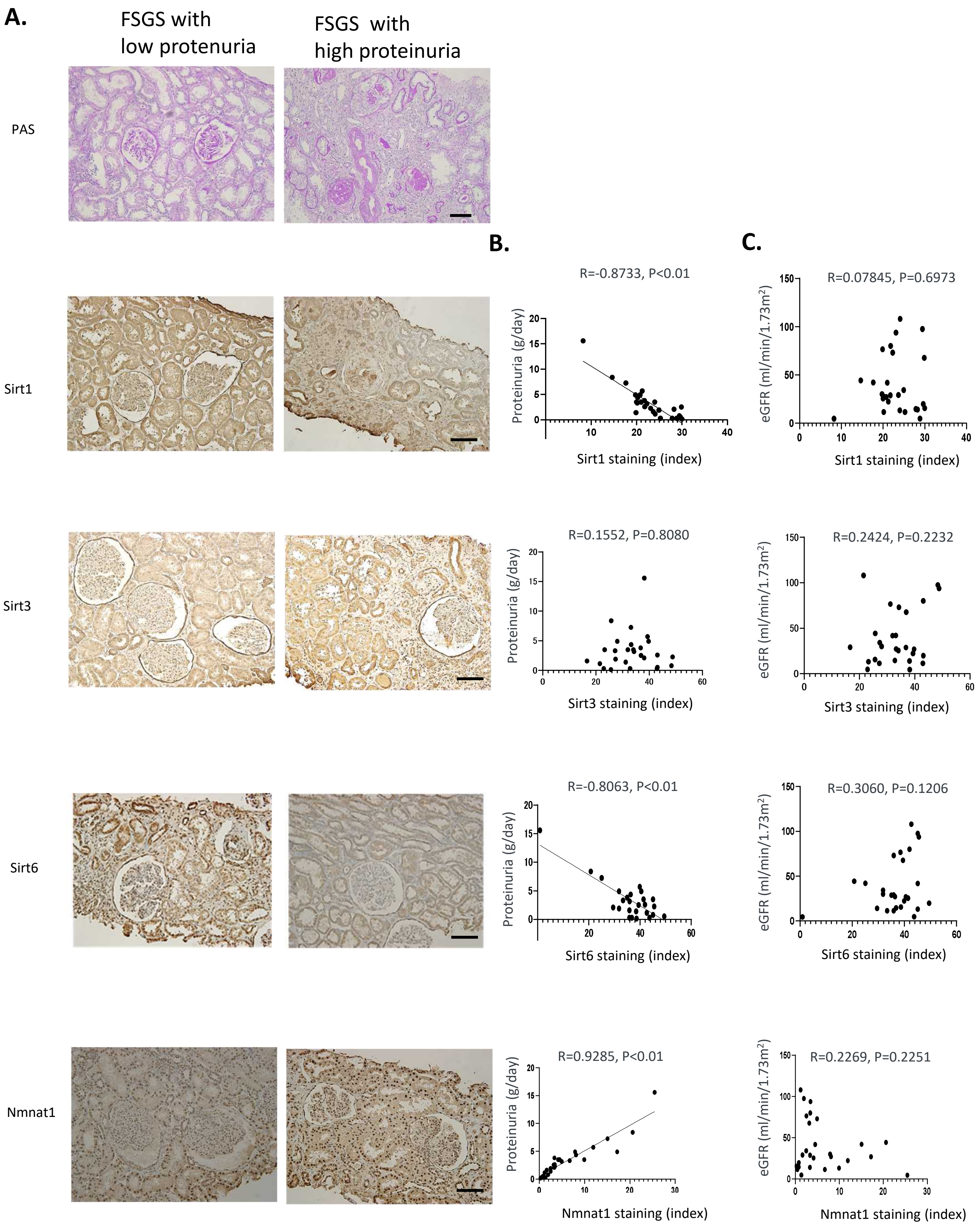


Supplementary Figure 1 , Hasegawa *et al.*

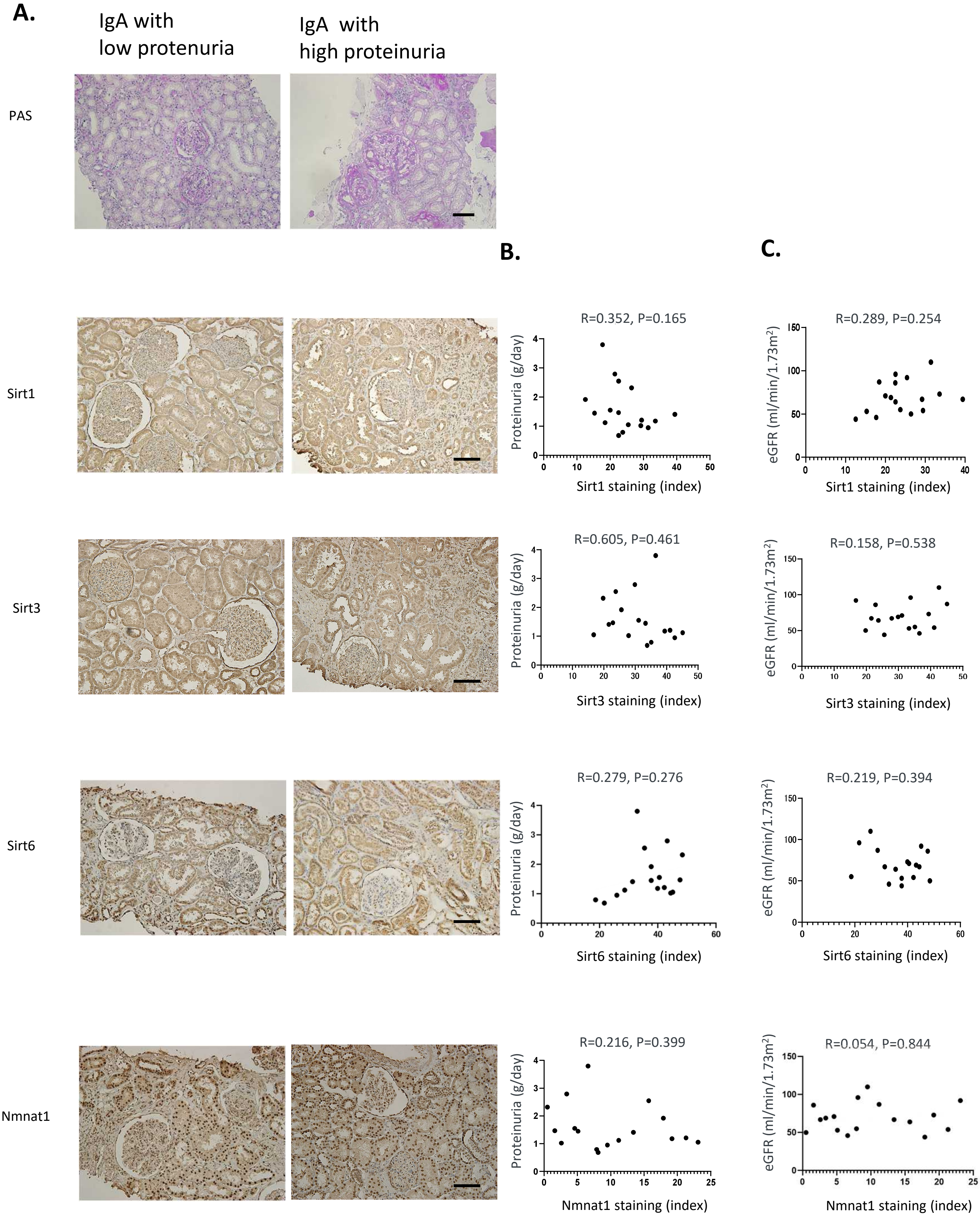


Supplementary Figure 2 , Hasegawa *et al.*

**A.**

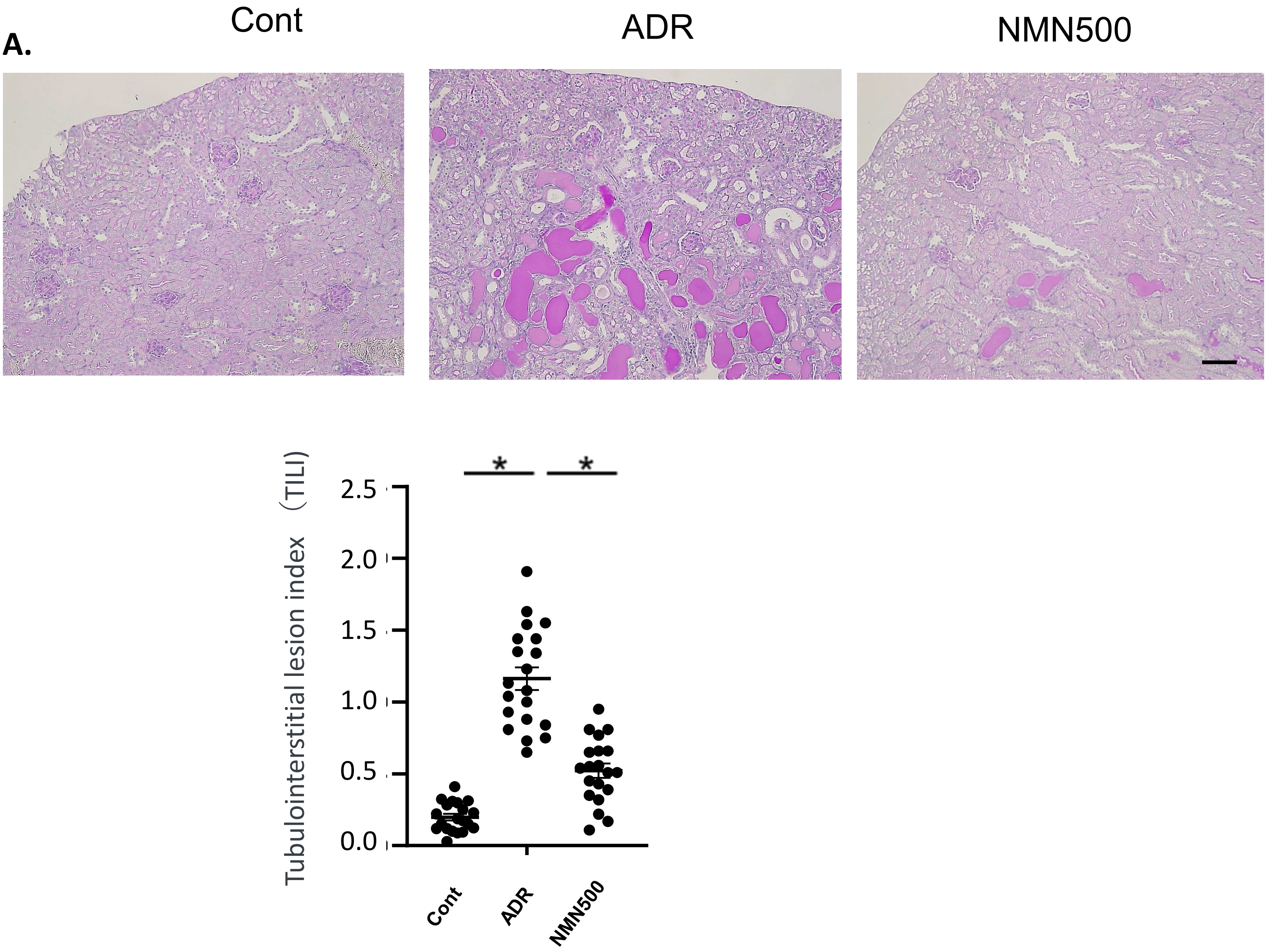


**B.**

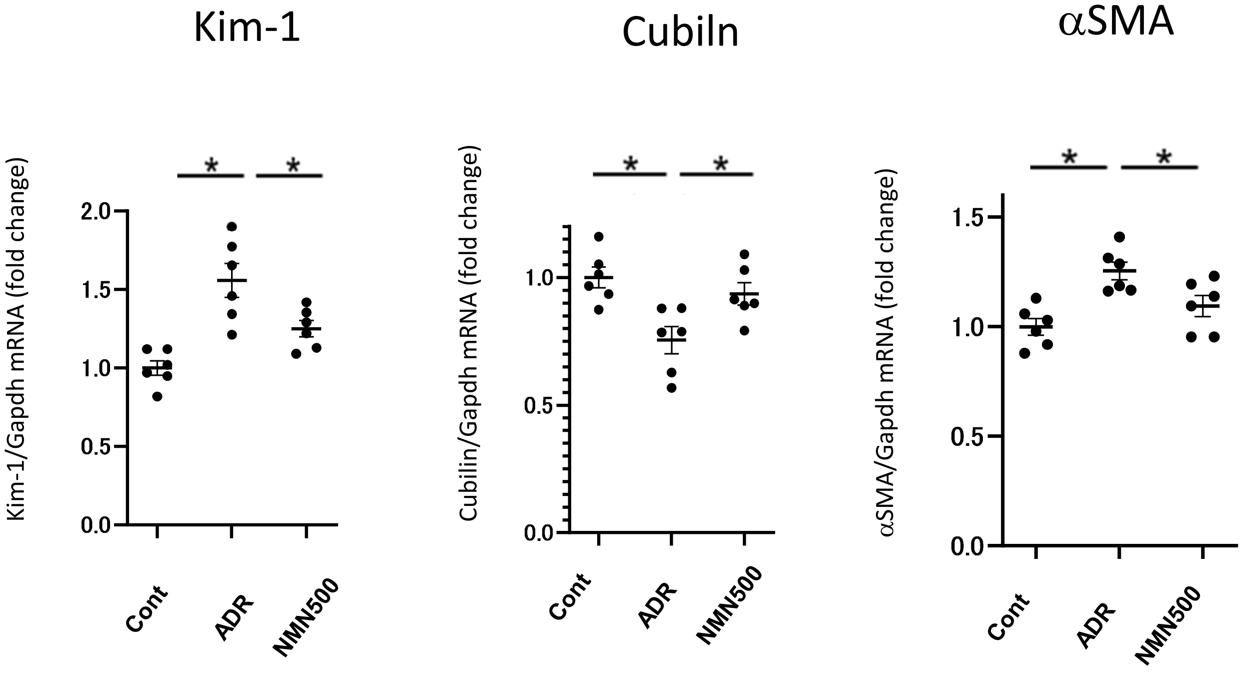


Supplementary Figure 3 , Hasegawa *et al.*

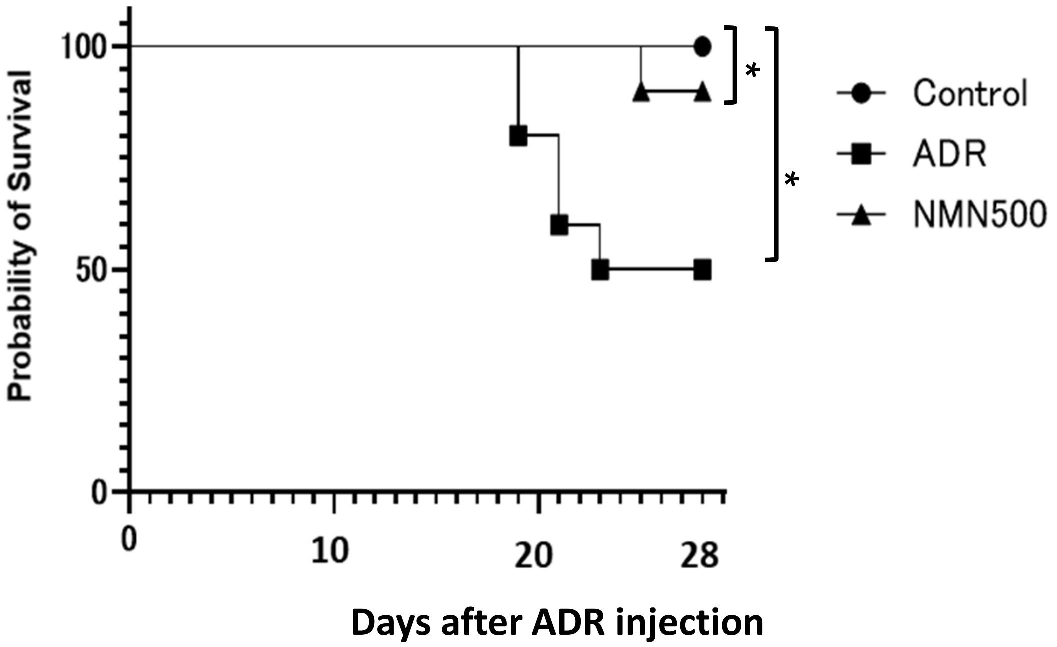


Supplementary Figure 4 , Hasegawa *et al.*

M U M U M U M U

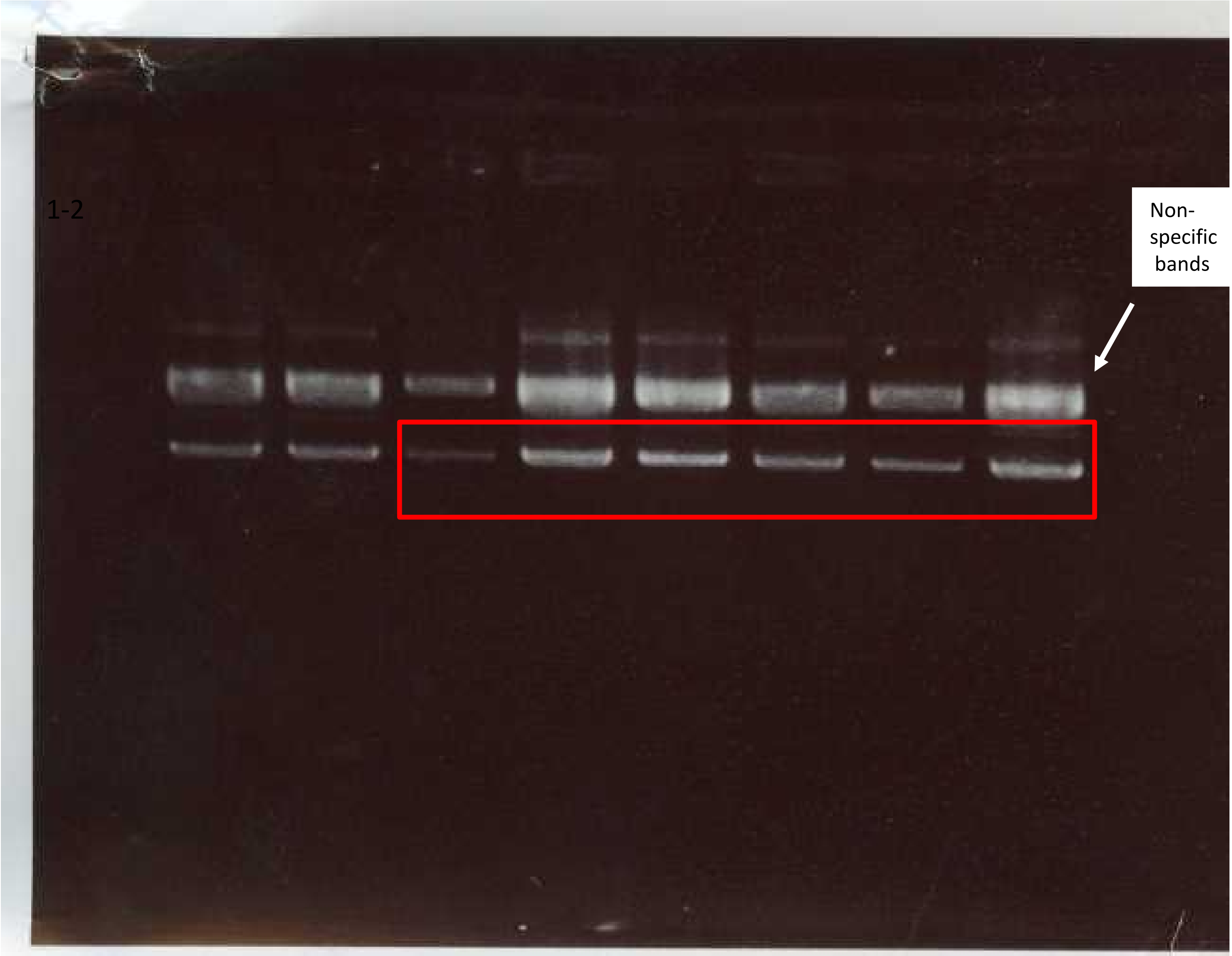


siRNA Cont Cont Cont Dnmt1

NMN (−) (−) (+)

ADR (−) (+)

Supplementary Figure 5 , Hasegawa *et al.*

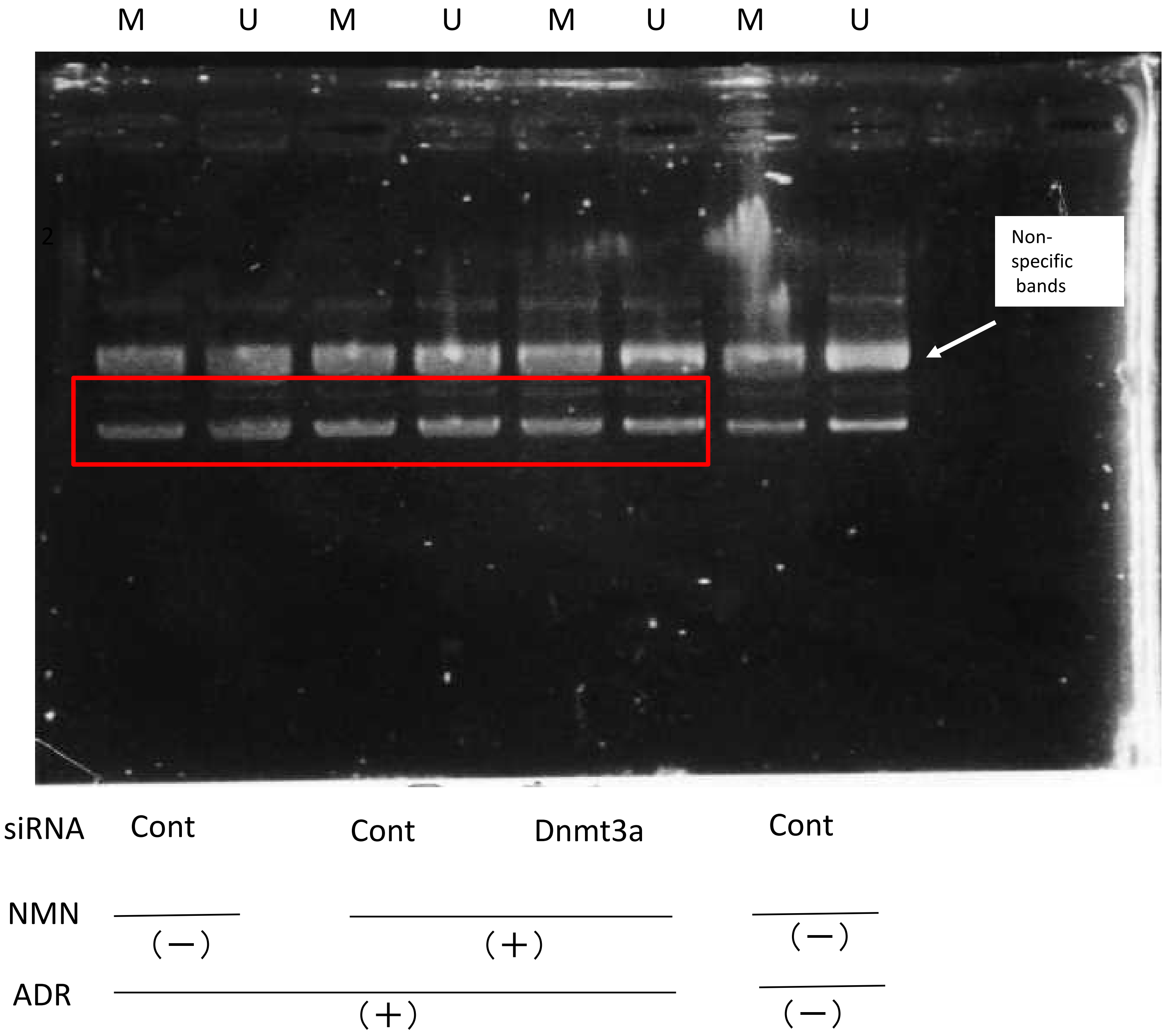


Supplementary Figure 6 , Hasegawa *et al.*

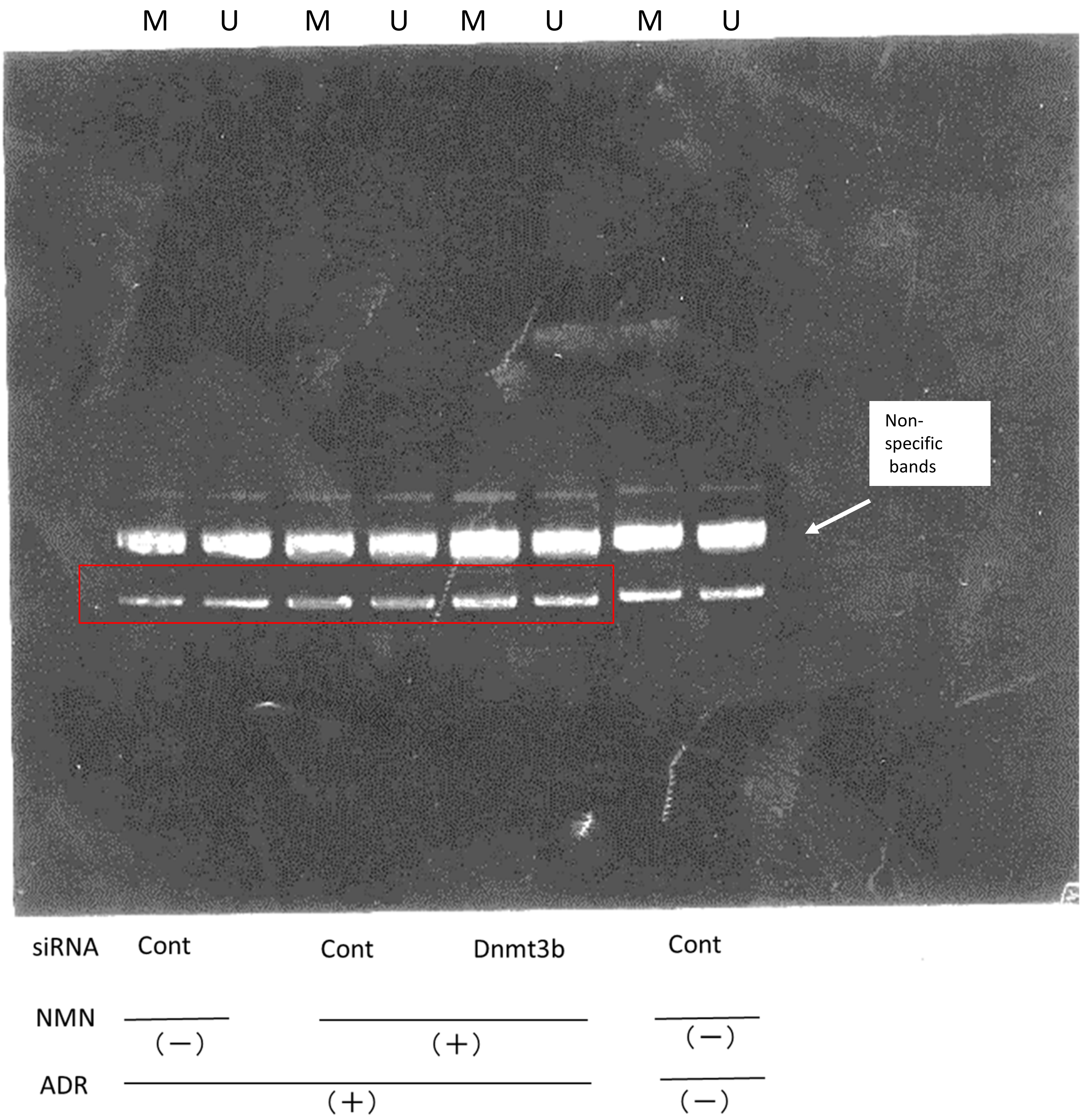


Supplementary Figure 7, Hasegawa *et al.*

**Supplementary Figure Legend**

**Supplementary Figure 1. Immunostaining in human renal biopsy specimens of focal segmental glomerulosclerosis (FSGS)**

**(A)** Representative photomicrographs of periodic acid–Schiff staining and immunostaining for Sirt1, Sirt3, Sirt6, and Nmnat1 in needle renal biopsy specimens of FSGS (sample names FSGS-5 and 11, Supplementary Table 1). Expression levels of Sirt1 and Sirt6 were decreased and levels of Nmnat1 were increased in patients with FSGS with heavy proteinuria compared with patients with FSGS with mild proteinuria. Bars; 50 nm. **(B)** Correlations between proteinuria and immunostaining for Sirt1, Sirt3, Sirt6, and Nmnat1 (n = 27). **(C)** Correlations between eGFR and immunostaining for Sirt1, Sirt3, Sirt6, and Nmnat1 (n = 27). Pearson's correlation analysis was used to calculate *r* and *P* values.

**Supplementary Figure 2. Immunostaining in human renal biopsy specimens of immunoglobulin (Ig)-A nephropathy**

**(A)** Representative photomicrographs of periodic acid–Schiff staining and immunostaining for Sirt1, Sirt3, Sirt6, and Nmnat1 in needle renal biopsy specimens of IgA nephropathy (sample names IgA-17 and IgA-2, Supplementary Table 2). (**B**) No differences in immunostaining intensities for Sirt1, Sirt3, Sirt6, or Nmnat1 were observed between samples from patients with

heavy proteinuria and low proteinuria. (**C)** No correlations were observed between eGFR and immunostaining intensities for Sirt1, Sirt3, Sirt6, or Nmnat1 (n = 17). Pearson's correlation analysis was used to calculate *r* and *P* values.

**Supplementary Figure 3. Nicotinamide mononucleotide ameliorates renal tubulointerstitial damages in adriamycin (ADR) nephropathy**

(**A)** Representative low-power field micrographs of renal histology samples at day 28 after ADR injection. Kidney sections were stained with periodic acid–Schiff. Mean tubulointerstitial lesion index scores for 20 representative sections are shown for each experimental group. (**B**) Real-time quantitative reverse transcription analysis of renal mRNA levels of Kim-1, cubilin, and α–smooth muscle actin (n = 6). Glyceraldehyde 3-phosphate dehydrogenase served as a control. All data are presented as the mean ± standard error of the mean. Horizontal bars indicate statistically significant differences between groups. $*P < 0.05$.

**Supplementary Figure 4. Nicotinamide mononucleotide (NMN) improves survival in adriamycin (ADR)-treated mice**

BALB/c mice were intravenously injected with ADR on day 0 as described in the methods section. Kaplan–Meier curves for each treatment group are shown (Cont, ADR, and NMN500; n = 30).

Differences between groups were assessed using the log-rank test.

**Supplementary Figures 5, 6, 7. Full-length gels from Figures 8B, 8C, and 8D**

Products of methylation-specific polymerase chain reaction. Bands of products included in Figures 8B, C, and D are highlighted by red boxes. Arrows indicate nonspecific bands.
